# Impact of policy measures targeting benzodiazepines and Benzodiazepine-related drugs in Lithuania: interrupted time series analysis

**DOI:** 10.1007/s00228-025-03992-7

**Published:** 2026-02-06

**Authors:** Tomas Lasys, Sharon C.M. Essink, Yared Santa-Ana-Tellez, Satu J. Siiskonen, Daniala L. Weir, Inge M. Zomerdijk, Rolf H.H. Groenwold, Marie L. De Bruin, Helga Gardarsdottir

**Affiliations:** 1 Division of Pharmacoepidemiology and Clinical Pharmacology, Utrecht Institute for Pharmaceutical Sciences (UIPS), Utrecht University, Utrecht, the Netherlands; 2 Department of Pharmacovigilance, Medicines Evaluation Board, Utrecht, the Netherlands; 3 School of Pharmacy, University of Waterloo, Waterloo, ON Canada; 4 Department of Clinical Epidemiology, Leiden University Medical Centre, Leiden, the Netherlands; 5 Department of Biomedical Data Sciences, Leiden University Medical Centre, Leiden, the Netherlands; 6 Department of Pharmaceutical Sciences, School of Health Sciences, University of Iceland, Reykjavik, Iceland; 7 Department of Clinical Pharmacy, University Medical Centre Utrecht, Utrecht, the Netherlands

**Keywords:** Benzodiazepines, Risk minimisation measures, Pharmacovigilance, Impact studies

## Abstract

**Purpose:**

Prescribing recommendations, communication programmes, and mandatory electronic prescribing were implemented in Lithuania to promote responsible use of benzodiazepines and benzodiazepine-related drugs (BZRDs). We assessed the impact of policy measures on benzodiazepine/BZRD prescribing patterns in Lithuania.

**Methods:**

We analysed utilisation of oral benzodiazepines/BZRDs in Lithuania from 2018 to 2024, using national prescription data and wholesale medicines data. Benzodiazepines/BZRDs included (1) anxiolytic benzodiazepines; (2) hypnotic benzodiazepines; and (3) BZRDs. The policy intervention period spanned from November 1, 2020, to July 1, 2021. We used ARIMA models to assess monthly incidence, prevalence, and long-term use prevalence (≥ 180 days) per 1,000 inhabitants. Besides, the numbers of defined daily doses (DDDs) prescribed and sold to pharmacies per 1,000 inhabitants were studied. We estimated baseline slopes, step changes after implementation, and changes in slopes after implementation.

**Results:**

In total, 717,590 patients received 6,974,059 prescriptions for benzodiazepines or BZRDs. Prior to implementing the policy measures, there were upward trends in incidence, prevalence, and long-term use prevalence across all classes. Following the implementation, monthly incidence stabilised and prevalence decelerated for anxiolytic benzodiazepines and BZRDs. The prevalence of hypnotic benzodiazepines showed a significant immediate reduction after implementation. Long-term use prevalence continued to increase for all benzodiazepine/BZRD classes after implementation of the policy.

Before the policy measures were implemented, monthly DDDs sold to pharmacies were gradually declining for anxiolytic benzodiazepines, but were stable for other classes. In contrast, monthly DDDs prescribed were increasing across all classes. Following the policy measures, immediate reductions in DDDs sold to pharmacies were observed, without changes in slopes. The prescribed and sold DDDs converged after implementing the policy measures.

**Conclusion:**

From a public health perspective, the policy measures implemented in 2020 and 2021 had only a limited impact on the prescribing patterns of benzodiazepines and BZRDs in Lithuania. They continued to be used long-term, highlighting the persistence of potentially irrational prescribing.

**Supplementary Information:**

The online version contains supplementary material available at 10.1007/s00228-025-03992-7.

## Introduction

Benzodiazepines and benzodiazepine-related drugs (BZRDs) are prescribed for various conditions, including anxiety, sleep, and seizure disorders, as well as agitation. They are also used as premedication before general anaesthesia and for sedation during minor procedures. However, use of these medicines is associated with harm, such as overdose, dependence, and mortality, especially in the elderly [1]. Despite these risks, benzodiazepines and, to a lesser extent, BZRDs are among the most commonly prescribed medicines worldwide [1–4].

Previous research indicates that benzodiazepine use is more than twice as prevalent in Lithuania compared to the other Baltic and Nordic countries [5, 6]. However, most cross-country comparison studies have assessed drug use based on Defined Daily Doses (DDDs) consumed per 1,000 inhabitants, limiting our ability to compare patient-level patterns. Moreover, the few studies that explored benzodiazepine user characteristics in Lithuania rely on small sample sizes, which may not be representative of the entire population [7, 8]. The Lithuanian government has estimated that approximately one quarter of inhabitants in Lithuania have used sedative and/or hypnotic medicines, including benzodiazepines, at least once in their lifetime, prompting the implementation of the policy measures to curb use [9].

In 2020 and 2021, the Lithuanian government introduced national guidelines, launched communication programmes, and provided general practitioner training sessions to address high and irrational use of benzodiazepines and BZRDs (Table 1) [9, 10]. A follow-up regulation mandated electronic or tamper-resistant prescriptions to improve transparency and reduce misuse [11]. However, the impact of these policy measures on benzodiazepine and BZRDs prescribing patterns, as well as the use of alternative pharmacological treatments, remains unclear. Evaluating the impact of policy measures is essential for informing future strategies aimed at optimising benzodiazepine and BZRD prescribing practices and reducing inappropriate use. This study aimed to assess the impact of the policy measures introduced in 2020–2021 on the prescribing patterns of benzodiazepines and BZRDs in Lithuania.

**Table 1:** Policy measures introduced for benzodiazepines and benzodiazepine-related drugs in Lithuania between 2020 to 2021 [9–11]

Policy measure	Details	Implementation date	Objectives
Prescribing recommendations	Benzodiazepines and BZRDs should be prescribed for a maximum of 4–12 weeks, depending on the indication; Guidelines for tapering or discontinuation in long-term benzodiazepines and/or BZRD users were introduced; Non-pharmacological treatments should be prioritised before initiating benzodiazepines and BZRDs; Alternative treatments should be considered for persistent conditions previously managed with benzodiazepines and BZRDs; Highlighted that benzodiazepines and BZRDs are not recommended for specific populations, including pregnant/lactating patients, individuals over 65 years of age, those with severe renal, hepatic, or pulmonary impairment, and patients with a history of substance use disorders.	July 1, 2020 (published); November 1, 2020 (enforced)^A^	Promote rational prescribing; Reduce and prevent long-term use; Encourage safer treatment alternatives.
Communication programmes and general practitioner training sessions	Educating the public and healthcare professionals on rational benzodiazepine and BZRD use; Training to general practitioners on appropriate prescribing, reaching approximately half of the licensed general practitioners in Lithuania.	November 2020	Increase awareness and promote adherence to prescribing recommendations.
Mandatory electronic or tamper-resistant prescription	Mandating that benzodiazepines and BZRDs are prescribed exclusively through either the national electronic prescription system or tamper-resistant prescription pads.	July 1, 2021	Enhance transparency; Improve monitoring of prescribing patterns; Reduce falsified prescriptions.

^A^ The recommendations were issued as a Ministerial Order, a binding legal act obligating all licensed healthcare professionals and institutions in Lithuania to comply with its provisions.

BZRD, benzodiazepine-related drug.

## Methods

### Study design

To assess the impact of the policy measures introduced for benzodiazepines and BZRDs in Lithuania, we performed a drug utilisation study using national databases, covering the entire Lithuanian population. The study period was from January 1, 2018, to December 31, 2024.

### Data sources

Data were obtained via Lithuanian Health Data Reuse Pathway from the electronic prescription database, which is part of the Lithuanian Electronic Health Services and Collaboration Infrastructure Information System (ESPBI IS) [12, 13]. It captures comprehensive prescription data, including the prescription date, Anatomical Therapeutic Chemical (ATC) classification code, strength, quantity prescribed, treatment duration, and indication coded using International Classification of Diseases-Tenth Revision (ICD-10). Additionally, patient demographics (age, sex) and prescriber speciality are recorded. The electronic prescription database includes electronic prescriptions provided they are issued through the electronic prescription subsystem. The electronic prescription system was introduced in 2015. It has been mandatory to use in all community pharmacies since 2015 and was gradually implemented in all personal health care institutions, being mandatory since March 2018 [14].

Additionally, wholesale medicines data were analysed in this study, which provides information on the quantity of medicines sold to community pharmacies. These data are collected by the Lithuanian State Medicines Agency and are publicly available [15].

Lastly, data from the National Health Insurance database, provided by the National Data Agency, were used to define the number of inhabitants eligible for prescriptions. The monthly number of inhabitants was defined as the count of unique individuals with at least one day of valid health insurance coverage [12].

### Cohort definition and included benzodiazepines/BZRDs

Our main cohort consisted of all patients enrolled in the electronic prescription database with a prescription for benzodiazepines and/or BZRDs between January 1, 2018, and December 31, 2024. Relevant prescriptions were identified using level 5 ATC codes. Benzodiazepines and BZRDs were divided into three classes, based on ATC codes [16]: (1) anxiolytic benzodiazepines (N05BA), prescribed to treat a range of conditions including anxiety; (2) hypnotic benzodiazepines (N05CD), primarily prescribed to treat sleep disorders; (3) BZRDs (N05CF), primarily prescribed for short-term management of sleep disorders. Only oral benzodiazepines and BZRDs were included because these were the principal targets of the policy measures in Lithuania. Clonazepam (N03AE01) is indicated for epilepsy and was not targeted by the policy measures; therefore, it was not included in our main cohort.

To explore potential shifts in prescribing patterns, we also included data on oral alternative medicines prescribed for anxiety, mood disorders, and sleep disorders. These medicines were not part of the main cohort but were included for exploratory purposes and included: gabapentinoids (N02BF); antiepileptic benzodiazepines (N03AE); diazepines, oxazepines, thiazepines, and oxepines (N05AH); diphenylmethane derivatives (N05BB); carbamates (N05BC); azaspirodecanedione derivatives (N05BE); other anxiolytics (N05BX); melatonin receptor agonists (N05CH); non-selective monoamine reuptake inhibitors (N06AA); monoamine oxidase inhibitors, non-selective (N06AF); and monoamine oxidase A inhibitors (N06AG); selective serotonin reuptake inhibitors (SSRIs, N06AB); and other antidepressants (N06AX) [17, 18].

Only prescriptions with a valid patient ID were included, as prescriptions without a valid patient ID could not be linked to individual patients. We excluded prescriptions with erroneous treatment duration (treatment duration of 0 or > 360 days) and quantity values (missing strength or > 4 DDDs per day) that could not be corrected using prior prescription data or assumption of 1 DDD per day. This led to the exclusion of *n* = 10,608 prescriptions and *n* = 384 patients for our main cohort (supplementary material [Figure S1]).

### Policy measures

Because of the short duration between the implementation of several policy measures targeting benzodiazepines and BZRDs, we considered all policy measures targeting benzodiazepines and BZRDs as one combined intervention lasting from November 1, 2020, until July 1, 2021 (Table 1) [9–11].

### Drug utilisation measures

Treatment episodes were constructed by linking consecutive prescriptions for the same medicine. If prescriptions overlapped in duration, the overlapping days were added to the end of the treatment episode. However, if no new prescription was issued within 30 days after the end of the episode, the extension was capped at a maximum of 30 days.

Incidence was defined as the number of patients initiating treatment with a medicine of a given class within a particular month following a minimum of 180 days without use of the medicine of the same class, with the condition that patients have been enrolled in the database for at least 180 days. Prevalence referred to the total number of unique patients receiving at least one prescription for a medicine of a given class within a particular month. Long-term use was characterised as a treatment duration of at least 180 days, allowing treatment gaps of up to 30 days between prescriptions. In the supplementary material (Figure S2 and Figure S3), schematic overviews of the definitions of incidence, prevalence, and long-term use are provided.

Monthly incidence, prevalence, and long-term use prevalence were calculated by dividing the respective number of patients with at least one prescription within a month by the number of inhabitants in 1,000s for the corresponding month.

### Outcomes

Our primary outcomes included monthly incidence, prevalence, and long-term use prevalence for each benzodiazepine/BZRD class, expressed per 1,000 inhabitants with valid health insurance at the start of the month, based on electronic prescription data. Incidence and prevalence were assessed from January 1, 2018. Long-term use prevalence was only assessed from January 1, 2019, onwards to account for the time required to be classified as long-term use.

As a secondary outcome, monthly defined daily doses (DDDs) per 1,000 inhabitants with valid health insurance at the start of the month for each benzodiazepine/BZRD class were retrieved from both electronic prescription data and wholesale medicines data (Supplementary material [Table S2]).

Furthermore, we explored monthly incidences and prevalences of alternative medicines per 1,000 inhabitants with valid health insurance at the start of the month, as recorded in the electronic prescription database.

### Covariates

Patients were assessed by sex and age at the time of their first prescription within our study period (age groups: <18; 18–29; 30–44; 45–59; 60–74; ≥75 years).

Indications are directly linked to prescriptions in the electronic prescription database and were grouped into anxiety and related disorders (ICD-10 codes: F40*-F48*; F06.4); mood disorders (ICD codes: F30*-F39*; F06.3); sleep disorders (ICD codes: G47*; F51*); other mental disorders (ICD codes: F00*-F06.2; F06.5-F29*; F50*; F52*-F99*); other disorders, as these are most relevant to assess the impact of the policy measures.

We assessed prescriber speciality using the following categories: general practitioner, psychiatrist (including child and adolescent psychiatrist), internal medicine physician, neurologist, other speciality, and unknown. Prescribers with multiple specialities were included in all relevant categories.

### Data analysis

Descriptive statistics were used to describe patient and prescription characteristics. Continuous variables were described using the median with first (Q1) and third (Q3) quartiles. For categorical variables, frequencies and proportions were reported.

To evaluate the possible impact of the policy measures, we fitted Autoregressive Integrated Moving Average (ARIMA) models using the forecast package in R for incidence, prevalence, and long-term use prevalence. ARIMA models allow an examination of changes in prescribing patterns while accounting for autocorrelation between consecutive monthly observations and seasonality [19]. To account for variation in the number of working days per month, incidence, prevalence, and long-term use prevalence were standardised to the number of working days. Two periods were constructed, a pre-policy period (January 1, 2018 – November 1, 2020) and post-policy period (July 1, 2021 - December 31, 2024).

The regression structure of the ARIMA models included: (1) a baseline slope, representing the monthly change in the outcome prior to the implementation of the policy measures; (2) a step change, capturing any immediate change in the outcome at the time of implementation of the policy measures; and (3) a change in slope, reflecting the additional monthly change in the outcome after implementation of the policy measures. To prioritise parsimony, the underlying time series structure was determined by selecting ARIMA models with the lowest Bayesian Information Criterion (BIC), using the auto.arima() function. The model search was constrained to a maximum of two for the autoregressive (p), differencing (d), and moving average (q) parameters. The inclusion of a seasonal component was also guided by BIC, with seasonal terms retained only when they improved model fit.

The analyses of the primary outcomes were also stratified by sex, age groups, indications for prescription, and prescriber speciality. For analyses stratified by sex and age groups, we used monthly counts of inhabitants similarly stratified based on sex and age to have subgroup-specific calculations.

We also examined the monthly proportions of incident and long-term users among all prevalent benzodiazepine/BZRD users to provide further insight into patterns over time. These were calculated by dividing the number of incident or long-term users by the total number of prevalent users in a given month.

To assess potential benzodiazepine and BZRD use not captured by electronic prescriptions, we applied ARIMA models on monthly DDDs sold to pharmacies and prescribed per 1,000 inhabitants, using the same approach as for our primary outcomes.

Data were prepared and analysed using Palantir Foundry (version 6.413.42) and R (version 4.4.2 using RStudio 2025.05.0). Data cleaning and analysis was performed and double checked by two researchers (T.L. and S.C.M.E.).

## Results

### Patient characteristics

A total of 717,590 patients received at least one prescription for a benzodiazepine or BZRD between 2018 and 2024. Among these, 610,168 patients were prescribed anxiolytic benzodiazepines, 86,302 hypnotic benzodiazepines, and 235,525 BZRDs. The patients were predominantly female (*n* = 499,668, 69.6%; Table 2). The median age of patients was 63 years (Q1-Q3: 50–74 years), with the majority of patients aged 45 years or older (82.3%). Patient characteristics were similar across the different benzodiazepine/BZRD classes.

**Table 2:** Characteristics of patients with a prescription for benzodiazepines and benzodiazepine-related drugs in Lithuania between January 1, 2018, and December 31, 2024

	All benzodiazepines/BZRDs,n = 717,590	Anxiolytic benzodiazepines (N05BA),n = 610,168	Hypnotic benzodiazepines (N05CD),n = 86,302	Benzodiazepinerelated drugs (N05CF),n = 235,525
**Sex, n (%)**				
Male	217,922 (30.4)	174,094 (28.6)	25,778 (29.9)	73,859 (31.4)
Female	499,668 (69.6)	436,074 (71.5)	60,524 (70.1)	161,666 (68.6)
Age in years, median (Q1-Q3)	63 (50–74)	63 (50–75)	66 (56–76)	64 (53–75)
**Age group, n (%)**				
<18 years	3,601 (0.5)	3,087 (0.5)	494 (0.6)	200 (0.1)
18–29 years	33,294 (4.6)	28,767 (4.7)	1,761 (2.0)	7,771 (3.3)
30–44 years	89,930 (12.5)	76,825 (12.6)	6,518 (7.6)	24,468 (10.4)
45–59 years	180,123 (25.1)	148,972 (24.4)	20,043 (23.2)	59,363 (25.2)
60–74 years	235,979 (32.9)	198,665 (32.6)	32,053 (37.1)	84,507 (35.9)
≥75 years	174,663 (24.3)	153,852 (25.2)	25,433 (29.5)	59,216 (25.1)
**Long treatment duration, n (%)**				
Use ≥ 90 days	149,352 (20.8)	119,880 (19.6)	14,288 (16.6)	36,124 (15.3)
Use ≥ 180 days	89,604 (12.5)	71,112 (11.7)	7,895 (9.1)	20,700 (8.8)
Use ≥ 365 days	50,581 (7.0)	40,077 (6.6)	3,537 (4.1)	11,046 (4.7)

Demographic characteristics (age and sex) were determined at the time of the first prescription within the specific benzodiazepine/BZRD class during the study period, while long treatment duration (≥ 90, ≥ 180, and ≥ 365 days) was assessed over the entire study period. Patients who received prescriptions in multiple benzodiazepine/BZRD classes were included in each respective class but counted only once in the overall ‘All benzodiazepines/BZRDs’ class.Q1, first quartile; Q3, third quartile.

### Benzodiazepine and BZRD prescription characteristics

In total, 6,974,059 benzodiazepine and BZRD prescriptions were issued during the study period (Table 3). Anxiolytic benzodiazepines was the most prevalent subgroup, accounting for almost three quarter of benzodiazepine/BZRDs prescriptions.

Sleep disorders and anxiety and related disorders were the most common indications for benzodiazepine and BZRD prescriptions, together accounting for approximately half of all use (27.7% and 25.3%, respectively; Table 3). The proportion of prescriptions for sleep disorders ranged from 19.5% for anxiolytic benzodiazepines to 51.3% for hypnotic benzodiazepines. In contrast, the proportion of all prescriptions for anxiety and related disorders was the highest for anxiolytic benzodiazepines (28.9%). The remaining prescriptions for benzodiazepines/BZRDs were issued for a broad range of other conditions, including conditions outside approved indications for benzodiazepine/BZRD use. Examples of non-approved indications are mood disorders (19.2%) and non-psychiatric conditions such as hypertensive heart disease (6.2%) or other cerebrovascular diseases (~ 1%) (Supplementary material [Table S3]).

General practitioners were the predominant prescribers, accounting for 57.0% of all benzodiazepine/BZRD prescriptions, followed by psychiatrists at 34.8%. The remaining prescriptions were issued by other specialists, including internal medicine physicians (9.7%) and neurologists (1.5%, Table 3). Prescriber speciality showed no major differences across the benzodiazepine/BZRD classes.

**Table 3:** Electronic prescription characteristics for benzodiazepines and benzodiazepine-related drugs issued in Lithuania between January 1, 2018, and December 31, 2024

	All benzodiazepines/BZRDs, *n* = 6,974,059	Anxiolytic benzodiazepines (N05BA), *n* = 5,142,266	Hypnotic benzodiazepines (N05CD), *n* = 470,608	Benzodiazepine-related drugs (N05CF), *n* = 1,361,185
**Indication for prescription, n (%)**				
Sleep disorders	1,928,455 (27.7)	1,001,721 (19.5)	241,317 (51.3)	685,417 (50.4)
Anxiety and related disorders	1,766,797 (25.3)	1,485,114 (28.9)	67,407 (14.3)	214,276 (15.7)
Mood disorders	1,336,755 (19.2)	1,047,704 (20.4)	73,224 (15.6)	215,827 (15.9)
Other mental disorders	866,387 (12.4)	730,186 (14.2)	34,237 (7.3)	101,964 (7.5)
Other disorders	1,075,665 (15.4)	877,541 (17.1)	54,423 (11.6)	143,701 (10.6)
**Prescriber speciality, n (%)**				
General practitioner	3,978,022 (57.0)	2,851,476 (55.5)	292,156 (62.1)	834,390 (61.3)
Psychiatrist	2,425,678 (34.8)	1,874,135 (36.4)	140,778 (29.9)	410,765 (30.2)
Internal medicine physician	676,511 (9.7)	498,090 (9.7)	49,674 (10.6)	128,747 (9.5)
Neurologist	103,115 (1.5)	75,091 (1.5)	5,484 (1.2)	22,540 (1.7)
Other speciality	246,464 (3.5)	180,295 (3.5)	17,096 (3.6)	49,073 (3.6)
Unknown	24,856 (0.4)	19,495 (0.4)	1,248 (0.3)	4,113 (0.3)

For each prescription, the indication was recorded as a mandatory field.Prescribers with multiple specialities were included in all relevant categories, resulting in sum of proportions exceeding 100%.

### Benzodiazepine/BZRD prescribing patterns

**Fig. 1:**
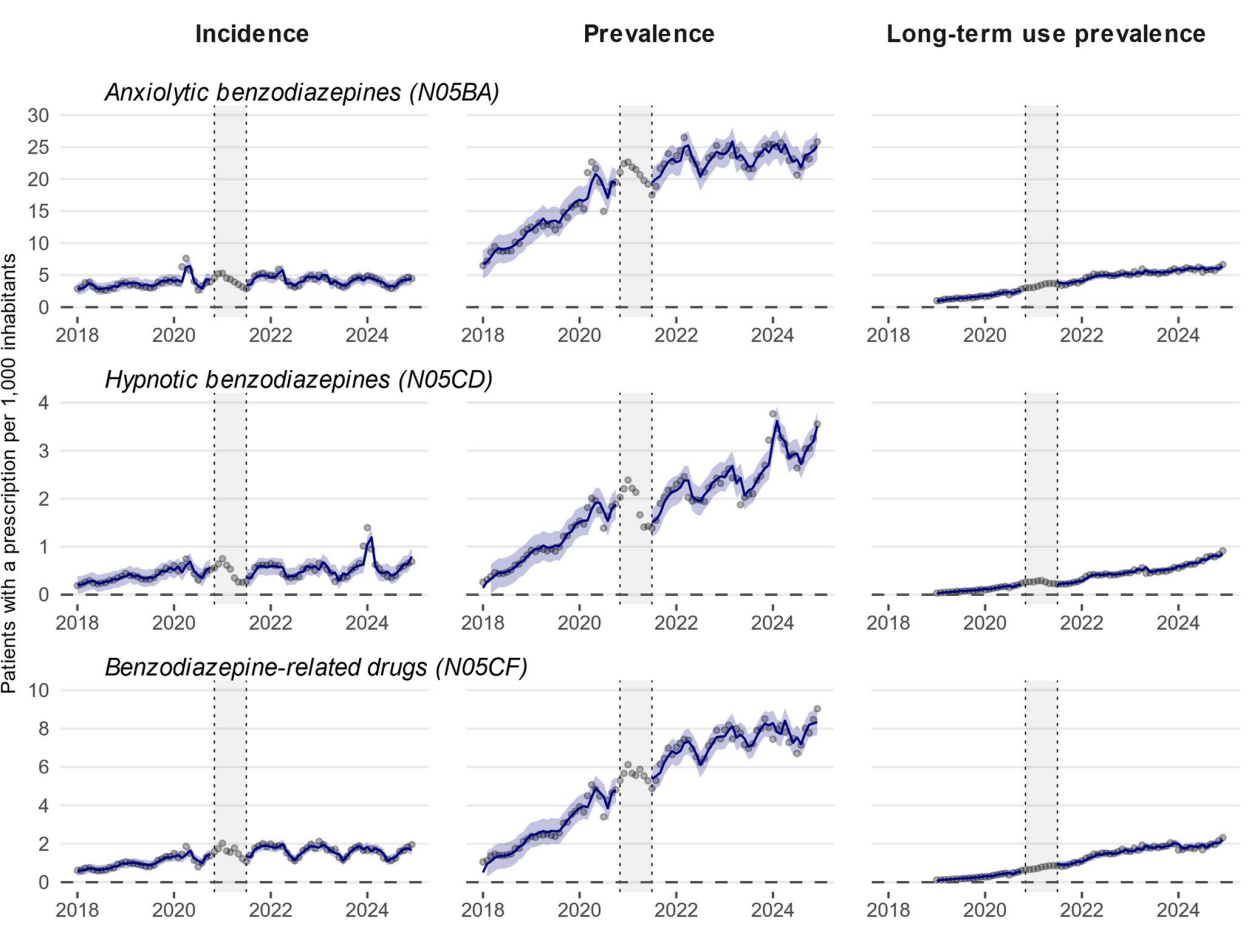
Monthly trends in incidence, prevalence, and long-term ^A^ use prevalence of benzodiazepines and benzodiazepine-related drugs in Lithuania from January 1, 2018, to December 31, 2024, based on electronic prescriptions. Lines represent modelled estimates with their 95% confidence intervals derived from ARIMA models, while points indicate monthly rates based on observed data in electronic prescription records. Incidence was defined as the number of patients initiating treatment with a medicine of a given class within a particular month following a minimum of 180 days without any prescription for a medicine of the same class, with the condition that patients have been enrolled in the database for at least 180 days. Prevalence referred to the number of unique patients receiving at least one prescription for a medicine of a given class within a particular month. Long-term use was characterised as a treatment duration of at least 180 days, allowing treatment gaps of up to 30 days between prescriptions. ^A^ Long-term use was only assessed from January 1, 2019, onwards

Prior to the introduction of the policy measures, there was an upward trend in incidence across all benzodiazepine/BZRD classes, with a baseline slope ranging from 0.011 [95% CI: 0.004 to 0.018] for hypnotic benzodiazepines to 0.055 [95% CI: 0.025 to 0.086] for anxiolytic benzodiazepines (Table 4; Fig. 1). Following the introduction of the policy measures, no immediate impact was observed, reflected by confidence intervals of the step change crossing 0 for all classes. However, a decline in the monthly trend was observed for anxiolytic benzodiazepines (change in slope: −0.073 [95% CI: −0.115 to −0.032]) and BZRDs (change in slope: −0.031 [95% CI: −0.040 to −0.022]). These decreases were comparable in magnitude to the previously increasing baseline slopes, indicating that incidence stabilised after the introduction of policy measures. No statistically significant monthly changes were detected for incidence of hypnotic benzodiazepines.

Prior to the implementation of policy measures, the prevalence of all benzodiazepine/BZRD classes was increasing, and at a faster rate than the incidence, as reflected by the baseline slopes (Table 4; Fig. 1). This suggests an increase in the average duration of use over time. Following the policy implementation, a small immediate reduction in prevalence was observed for hypnotic benzodiazepines (step change: −0.676 [95% CI: −1.153 to −0.200]), but no subsequent changes in monthly trend were detected. This indicates that, after a brief immediate drop in prevalence, prevalence continued to increase at the same pace as before the implementation of the policy measures. For anxiolytic benzodiazepines and BZRDs, no immediate effects were observed, but the upward trends in prevalence reduced after the implementation of policy measures (change in slope: −0.365 [95% CI: −0.473 to −0.257] for anxiolytic benzodiazepines; −0.092 [95% CI: −0.134 to −0.051] for BZRDs). The additional changes in prevalence were less pronounced than the baseline slope, thus, overall trends remained upward. This suggests that prevalence continued to increase, though at a slower rate, after the policy measures were introduced.

Analysis of long-term use prevalence revealed increasing trends prior to the implementation of the policy measures across all benzodiazepine/BZRD classes (Table 4; Fig. 1). Following the implementation, no significant changes were detected in either the immediate level (i.e., step change) or the subsequent trend (i.e., change in slope).

**Table 4:** ARIMA model estimates of monthly benzodiazepine and benzodiazepine-related drug prescribing patterns in Lithuania from January 1, 2018, to December 31, 2024, based on electronic prescription data

	Baseline slope [95% CI]		Step change [95% CI]		Change in slope [95% CI]	
**Incidence**						
Anxiolytic benzodiazepines (N05BA)	0.055 [0.025; 0.086]		−0.466 [−1.364; 0.432]		−0.073 [−0.115; −0.032]	
Hypnotic benzodiazepines (N05CD)	0.011 [0.004; 0.018]		−0.172 [−0.392; 0.048]		−0.008 [−0.017; 0.002]	
Benzodiazepine-related drugs (N05CF)	0.027 [0.021; 0.034]		0.020 [−0.169; 0.210]		−0.031 [−0.040; −0.022]	
**Prevalence**						
Anxiolytic benzodiazepines (N05BA)	0.418 [0.342; 0.495]		−1.860 [−4.078; 0.358]		−0.365 [−0.473; −0.257]	
Hypnotic benzodiazepines (N05CD)	0.057 [0.045; 0.068]		−0.676 [−1.153; −0.200]		−0.018 [−0.039; 0.002]	
Benzodiazepine related drugs (N05CF)	0.145 [0.122; 0.168]		0.016 [−0.903; 0.934]		−0.092 [−0.134; −0.05	
**Long-term use prevalence** ^**A**^						
Anxiolytic benzodiazepines (N05BA)	0.078 [0.045; 0.111]		0.695 [−0.047; 1.437]		−0.017 [−0.052; 0.019]	
Hypnotic benzodiazepines (N05CD)	0.010 [0.006; 0.014]		−0.105 [−0.237; 0.026]		0.005 [0; 0.010]	
Benzodiazepine-related drugs (N05CF)	0.025 [0.016; 0.035]		0.200 [−0.150; 0.551]		0.005 [−0.008; 0.017]	

The table presents (1) baseline slopes, representing monthly changes in the outcome prior to the implementation of the policy measures; (2) step changes, capturing any immediate change in the outcome at the time of implementation of the policy measures; and (3) changes in slopes, reflecting additional monthly changes in the outcome after implementation of the policy measures. Models were assessed for incidence, prevalence, and long-term use prevalence across the three benzodiazepine/BZRD classes. Incidence was defined as the number of patients initiating treatment with a medicine of a given class within a particular month following a minimum of 180 days without any prescription for a medicine of the same class, with the condition that patients have been enrolled in the database for at least 180 days. Prevalence referred to the total number of unique patients receiving at least one prescription for a medicine of a given class within a particular month. Long-term use was characterised as a treatment duration of at least 180 days, allowing treatment gaps of up to 30 days between prescriptions.

^A^ Long-term use was only assessed from January 1, 2019 onwards.

**Fig. 2:**
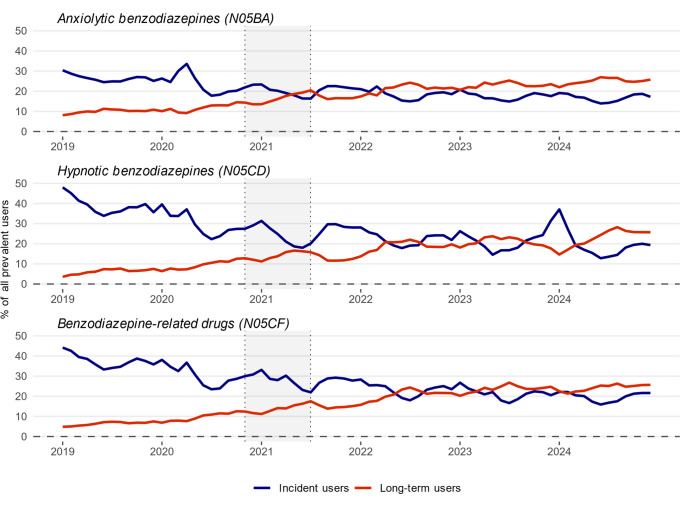
Proportion of incident and long-term users of benzodiazepines and benzodiazepine-related drugs among all prevalent users in Lithuania from January 1, 2019 to December 31, 2024, based on electronic prescriptions. Incidence was defined as the number of patients initiating treatment with a medicine of a given class within a particular month following a minimum of 180 days without use of medicine of the same class, with the condition that patients have been enrolled in the database for at least 180 days. Prevalence referred to the number of unique patients receiving at least one prescription for a medicine of a given class within a particular month. Long-term use was characterised as a treatment duration of at least 180 days, allowing treatment gaps of up to 30 days between prescriptions

Overall, the proportion of incident users among all benzodiazepine/BZRD users steadily declined during the study period (Fig. 2). In contrast, the proportion of long-term users continued to grow over time, accounting for around 20% of all users in the three benzodiazepine/BZRD classes after the introduction of the policy measures.

### Benzodiazepine/BZRD prescribing patterns stratified by (patient) subgroups

Analyses stratified by sex and age groups showed results similar to the main analyses without indicating any major differential effects for the impact of the policy measures on incidence, prevalence, and long-term use prevalence (Supplementary material [Tables S4-S5, Figures S4-S5]). Benzodiazepine/BZRD use remained high among women and, especially, patients above 60 years of age after implementation of the policy measures.

Analyses stratified by indication for benzodiazepine or BZRD use revealed minimal to no immediate impact of policy measures, followed by stabilising trends of incidence and slower increases in prevalence after the implementation of the policy measures in line with the main analyses (Supplementary material [Tables S6, Figure S6]). The ‘Other disorders’ group was the only indication group in which prevalence appeared to stabilise across all benzodiazepine/BZRD classes after the implementation of the policy measures.

Regarding prescriber speciality, incidence and prevalence of anxiolytic and hypnotic benzodiazepine prescribing by general practitioners and internal medicine physicians dropped immediately following the introduction of the policy measures (i.e., step changes), whereas this increased or was not affected in other prescriber specialities (Supplementary material [Table S7, Figure S7]). Subsequent monthly trends did not show any major long-term changes in prescribing trends or showed stabilising trends after the introduction of the policy measures across all benzodiazepine/BZRD classes and prescriber specialities. General practitioners remained the main prescribers of benzodiazepines and BZRDs after the implementation of the policy measures.

### Electronic prescription and wholesale medicines data on defined daily doses

**Fig. 3:**
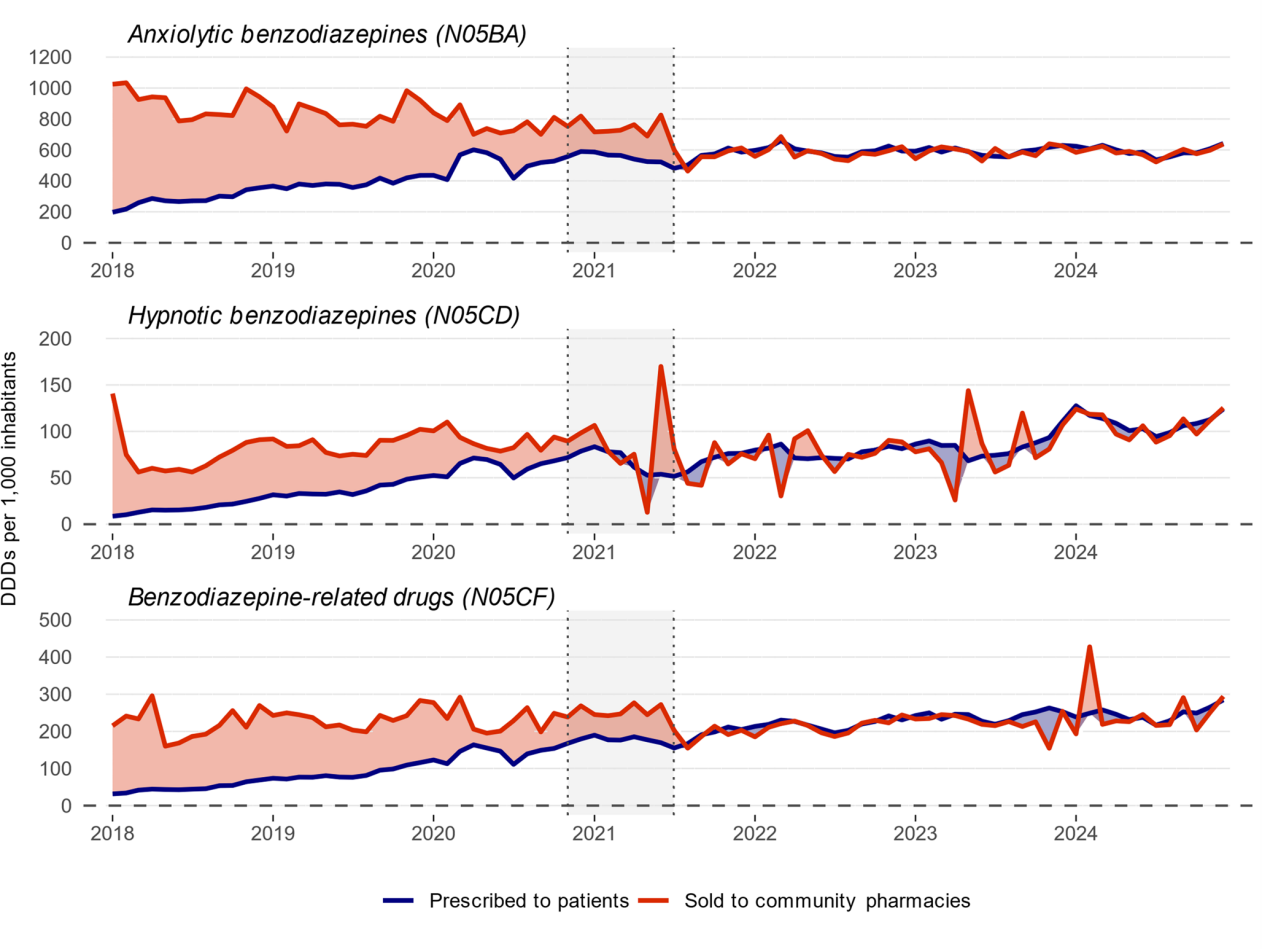
Monthly defined daily doses of benzodiazepines and benzodiazepine-related drugs prescribed electronically and sold to community pharmacies per 1,000 inhabitants based on electronic prescription data and wholesale medicines data in Lithuania from January 1, 2018, to December 31, 2024. The coloured area represents the discrepancy between DDDs prescribed to patients and DDDs sold to community pharmacies. A larger area indicates a greater difference, for example due to potential non-captured use in the electronic prescription data or stockpiling. DDD, defined daily dose

Before introducing the policy measures, monthly DDDs prescribed per 1,000 inhabitants increased across all benzodiazepine/BZRD classes, as indicated by the estimated baseline slopes derived from electronic prescription data (Table 5; Fig. 3). In contrast, during the same period, monthly DDDs sold to community pharmacies per 1,000 inhabitants were decreasing for anxiolytic benzodiazepines (baseline slope: −5.248, [95% CI: −6.723 to −3.772]), with no clear trends observed for hypnotic benzodiazepines and BZRDs.

Following the introduction of the policy measures, a significant immediate reduction in the monthly DDDs prescribed and DDDs sold to community pharmacies per 1,000 inhabitants was observed across nearly all benzodiazepine/BZRD classes. This immediate effect appeared more pronounced in the wholesale medicines data compared to electronic prescription data, e.g., a step change of −144.513, [95% CI: −187.618 to −101.408] versus − 58.858 [95% CI: −109.559 to −8.157] for anxiolytic benzodiazepines. However, the additional monthly changes (i.e., changes in slopes) were non-significant or of similar magnitude in a different direction from the baseline slopes, indicating stabilisation in DDDs prescribed and sold to community pharmacies after the implementation of the policy measures.

**Table 5:** ARIMA model estimates of monthly total defined daily doses for benzodiazepine and benzodiazepine-related drug in Lithuania from January 1, 2018, to December 31, 2024, based on electronic prescription data and wholesale medicines data

	Baseline slope [95% CI]	Step change [95% CI]	Change in slope [95% CI]
**DDDs prescribed per 1,000 inhabitants**			
Anxiolytic benzodiazepines (N05BA) Hypnotic benzodiazepines (N05CD) Benzodiazepine-related drugs (N05CF)	10.129 [8.423; 11.835] 2.023 [1.661; 2.384] 4.663 [4.016; 5.309]	−58.858 [−109.559; −8.157] −24.409 [−39.963; −8.855] −6.879 [−34.049; 20.291]	−9.485 [−11.839; −7.132] −0.731 [−1.376; −0.086] −2.881 [−4.039; −1.722]
**DDDs sold to community pharmacies per 1,000 inhabitants**			
Anxiolytic benzodiazepines (N05BA) Hypnotic benzodiazepines (N05CD) Benzodiazepine-related drugs (N05CF)	−5.248 [−6.723; −3.772] 0.576 [−0.075; 1.228] 0.545 [−0.671; 1.760]	−144.513 [−187.618; −101.408] −37.084 [−57.895; −16.272] −51.146 [−89.965; −12.327]	5.799 [3.847; 7.751] 0.589 [−0.217; 1.396] 0.980 [−0.524; 2.484]

The table presents (1) baseline slopes, representing monthly changes in the outcome prior to the implementation of the policy measures; (2) step changes, capturing any immediate change in the outcome at the time of implementation of the policy measures; and (3) changes in slopes, reflecting additional monthly changes in the outcome after implementation of the policy measures. Models were assessed for total DDDs across the three benzodiazepine/BZRD classes based on electronic prescription data and wholesale medicines data.

DDD, defined daily dose.

 Figure 3 indicates that wholesale medicines DDDs exceeded electronic prescribed DDDs before the introduction of the policy measures. The number of DDDs prescribed and sold to community pharmacies per 1,000 inhabitants converged after the introduction of the policy measures. 

### Alternative medicine prescribing patterns

In general, no notable changes in the prescribing of gabapentinoids (N02BF), antiepileptic benzodiazepines (N03AE), diazepines, oxazepanes, thiazepines, and oxepines (N05AH), azaspirodecanedione derivatives (N05BE), non-selective monoamine reuptake inhibitors (N06AA), SSRIs (N06AB), and other antidepressants (N06AX) were observed following the introduction of the policy measures, with changes in slopes being non-significant for incidence or indicating a slower rate of increase compared to baseline trends for prevalence (Supplementary material [Table S8]). The impact of the policy measures could not be assessed for the other alternative medicines since there were no electronic prescriptions prior to the introduction of the policy measures, or they were not registered in Lithuania.

## Discussion

In this study, we examined the impact of policy measures targeting high and irrational use of benzodiazepines and BZRDs in Lithuania, implemented between 2020 and 2021. Approximately one quarter of Lithuania’s inhabitants received at least one prescription for benzodiazepines or BZRDs between 2018 and 2024, which aligns with previous national estimates while providing a more precise assessment of current use based on population-level prescription data. Before the implementation of the policy measures, prescribing trends demonstrated increasing incidence, prevalence, and long-term use prevalence across all benzodiazepine/BZRD classes. We observed that, while the period following the introduction of the measures was accompanied by stabilisation of monthly incidence, prevalence continued to rise at a slower pace. Notably, long-term use prevalence continued to increase at a similar pace despite the implementation of the policy measures. We did not identify major changes in the prescribing of alternative medicines for anxiety, mood disorders, and sleep disorders, suggesting limited pharmacological substitution of benzodiazepines/BZRDs.

Irrational prescribing of benzodiazepines and BZRDs remains a concern in Lithuania. Prescribing recommendations explicitly recommend limiting benzodiazepine and BZRD use for a maximum of 4–12 weeks, depending on the indication, and provide tapering guidance for long-term users [10, 20–22]. Despite these recommendations, long-term use remained prevalent and continued to increase after 2021. Another finding is that the use of benzodiazepines and BZRDs is notably high among the older adults, with a median patient age of 63 (Q1-Q3: 50–74) years. Although prescribing recommendations highlight that these medicines should not be prescribed for people over 65 years, stratified analyses revealed no differential impact of the policy measures by age groups, with (long-term use) prevalence remaining highest among patients older than 60 years [1, 10]. These findings are particularly alarming given the well-documented risks associated with long-term use and use in the elderly, including abuse, dependence, risk of falls, and cognitive decline [1, 23, 24]. Studies conducted in other countries also reported that long-term use of benzodiazepines and BZRDs remains high, especially among the elderly, even following the implementation of targeted policies, similarly to our results [25–28].

Furthermore, published recommendations explicitly state that benzodiazepines and BZRDs should be prescribed only for approved indications, but we found that they continue to be used outside their approved indications, including for cardiovascular and mood disorders [10]. Encouragingly, our analysis stratified by indication suggests that the prevalence of benzodiazepines and BZRDs stabilised for the ‘Other disorders’ group, that predominantly covered use outside approved indications. This suggests that the policy measures might have been partially effective in curbing use outside approved indications. Nevertheless, use of benzodiazepines and BZRDs for non-approved indications appears to persist and has been reported across different healthcare systems, suggesting that this phenomenon is not unique to Lithuania [29, 30].

With regard to prescriber characteristics, general practitioners remained the main prescribers of benzodiazepines/BZRDs in Lithuania following the implementation of the policy measures. The introduction of these measures appeared to have caused an immediate reduction in prescribing of these medicines by general practitioners and internal medicine physicians. However, prescribing patterns subsequently stabilised after the implementation of the policy measures for these specialities, suggesting no major long-lasting effect.

Data on wholesale medicine sales to community pharmacies offered additional context to support and interpret findings from electronic prescription records. First, high monthly DDDs sold to community pharmacies prior to the policy measures suggest a greater extent of potentially inappropriate benzodiazepine or BZRD prescribing than is captured in the electronic prescription database. Second, the number of DDDs sold to community pharmacies and DDDs prescribed to patients converged following the implementation of the policy measures, suggesting improved data capture by the electronic prescription system. Minor misalignments and occasional spikes in DDDs sold to pharmacies are likely reflecting primary non-adherence (unfilled prescriptions) and possible stockpiling behaviour.

Furthermore, analysis of monthly DDDs of benzodiazepines and BZRDs sold to community pharmacies indicated a small immediate decrease, which suggests slight improvements in rational benzodiazepine and BZRDs use. We observed that the defined daily doses (DDDs) per 1,000 inhabitants for anxiolytic benzodiazepines was already declining prior to the implementation of the policy measures, with no clear trend for other benzodiazepine/BZRD classes. Across all classes, a moderate immediate drop in DDDs sold to community pharmacies was detected following the implementation, but the rates remained stable thereafter, indicating no sustained effect.

Discrepancies between estimated drug use from different data sources likely reflect the transition from paper to electronic prescriptions. Lithuania’s electronic prescription database was introduced in 2015 for all medicines and implemented gradually. Although it was made mandatory in all personal health care institutions by legislation in 2018, full implementation progressed gradually due to technical and logistical challenges, even after the legislation was formally implemented [14]. Government reports suggested faster transfer to electronic prescriptions for reimbursable medicines, whereas benzodiazepines and BZRDs are predominantly purchased out-of-pocket [9]. Thus, paper prescriptions for benzodiazepines and BZRDs remained relatively common after 2018, which led to the decision to mandate that these medicines be prescribed exclusively through either the national electronic prescription system or tamper-resistant prescription pads. Paper prescriptions for benzodiazepines and BZRDs remained available through special prescription pads even after the implementation of the policy measures. The convergence between electronic prescription data and wholesale medicines data supports the notion that electronic prescribing of benzodiazepines/BZRDs has become the new standard after July 2021. This indicates that the policy measures effectively established the electronic prescription system as the main channel for benzodiazepine/BZRD prescribing. Our study combining wholesale medicines and electronic prescription data suggests that the Lithuanian electronic prescription database is a reliable tool for monitoring trends of benzodiazepine and BZRDs use and evaluating policy outcomes in Lithuania in future.

The limited effectiveness of the Lithuanian policy measures aligns with findings from other countries. First, several measures for benzodiazepines and BZRDs were implemented in the Nordic countries, but a drug utilisation study over a period of almost 20 years observed that no single intervention has appeared to alter use patterns significantly [31]. Similarly, in the Netherlands, benzodiazepine (long-term) use remained stable following recommendations to only prescribe benzodiazepines for short-term [25]. Special prescription pads introduced in France have shown mixed success on impacting the benzodiazepine and BZRD use patterns. Some studies suggest more substantial impact for restrictions related to reimbursement status, but in Lithuania only selected benzodiazepines are reimbursed for limited number of conditions, including malignant neoplasms, epilepsy and some additional neurological disorders [20, 21, 32–34].

A strength of this study is the combination of electronic prescription data and wholesale medicines data, both providing national coverage. This approach allowed us to capture prescribing patterns more accurately, enhancing the validity of our findings. Moreover, the Lithuanian electronic prescribing system requires the entry of indications for the use of medicines, which is directly linked with a prescription. We also performed stratified analyses, which provided insights into effects across different (patient) subgroups.

However, limitations remain in our study. As stated before, electronic prescribing was made mandatory by legislation in 2018 in Lithuania, which could have caused underestimation of prevalent and long-term users, especially in the first years of our study period. Besides, some prevalent users could have been misclassified as incident users due to the transition from paper prescriptions to electronic prescriptions. Still, comparison with wholesale medicines data suggests that data quality and completeness of the electronic prescription system for benzodiazepines/BZRDs improved markedly after 2021. The indication for prescribing benzodiazepines/BZRDs is a mandatory field for electronic prescriptions, but might be prone to potential input errors by prescribers. In Lithuania, medicines are only reimbursed if they are prescribed for certain approved indications and such errors are sometimes resolved at the pharmacy level. However, as benzodiazepines/BZRDs are only rarely reimbursed, this corrective step could be applied less frequently. Furthermore, the wholesale data on medicines did not permit patient-level analysis, limiting our ability to assess individual use patterns. Lastly, the potential influence of the COVID-19 pandemic on benzodiazepine/BZRD prescribing trends cannot be entirely ruled out, as the pandemic period overlapped with the policy intervention period. Visual inspection of the data suggested at most a limited effect, characterized by a short-term increase in incidence, prevalence, and prescribed DDDs at the beginning of 2020, while no clear changes were observed in DDDs sold to pharmacies. Similar findings were reported in Estonia, where only a transient increase in benzodiazepine/BZRD use was observed following the onset of the pandemic [30].To curb irrational prescribing practices, understanding the underlying reasons for prescribing benzodiazepines and BZRDs in Lithuania is essential. Future research should explore factors influencing long-term use, prescribing in the elderly, and prescribing for non-approved indications. Moreover, studies should examine the conditions for which benzodiazepines and BZRDs are prescribed, with a focus on identifying non-pharmacological or safer pharmacological alternatives [35]. By assessing treatment trajectories for cohorts based on medical conditions, underutilised alternatives could be revealed and promoted. This would support the development of clearer guidelines and more targeted policy interventions, helping both physicians and patients make safer, evidence-based treatment decisions.

## Conclusion

The policy measures implemented in 2020 and 2021 had only a limited impact on the prescribing patterns of benzodiazepines and BZRDs in Lithuania. These medicines continued to be prescribed frequently and long-term, often off-label, and prescribing is particularly prevalent among the elderly. Our results highlight persistent challenges with potentially irrational use of benzodiazepines/BZRDs in Lithuania. While the policy measures fell short in addressing the key clinical concerns, they successfully accelerate the adoption of electronic prescribing of benzodiazepines/BZRDs. Future interventions might include deprescribing support and promotion of safe and effective treatment alternatives.

## Supplementary Information

Below is the link to the electronic supplementary material.

Supplementary Material 1 (9.82 MB)

## Data Availability

Data was obtained from State Data Agency (Statistics Lithuania) following the Lithuanian Law for Health Data Reuse. The authors are not allowed to share raw data in the public domain, but researchers and other persons can apply to access this data by submitting an application at https://duomenys.stat.gov.lt/health-data/[12]. The scripts used for data transformations and analysis will be published in an open GitHub repository upon publication of the manuscript (https://doi.org/10.17605/OSF.IO/FJT8K) [36].
