# Supplementary material for: Impact of policy measures targeting benzodiazepines and Benzodiazepine-related drugs in Lithuania: interrupted time series analysis
Source: Eur J Clin Pharmacol. 2026 Feb 6;82(3):68. doi: 10.1007/s00228-025-03992-7 (PMC12881020; doi:10.1007/s00228-025-03992-7)
Supplement: Supplementary file 1 — Supplementary Material 1 (9.82 MB) [file 228_2025_3992_MOESM1_ESM.docx]

**Impact of Policy Measures Targeting Benzodiazepines and Benzodiazepine-related Drugs in Lithuania; Interrupted Time Series Analysis**

Authors: Tomas Lasys^1^*, Sharon C.M. Essink^1,2^*, Yared Santa-Ana-Tellez^1^, Satu J. Siiskonen^1^, Daniala L. Weir^1,3^, Inge M. Zomerdijk^2^, Rolf H.H. Groenwold^4,5^, Marie L. De Bruin^1^, Helga Gardarsdottir^1,6,7^

^1^ Division of Pharmacoepidemiology and Clinical Pharmacology, Utrecht Institute for Pharmaceutical Sciences (UIPS), Utrecht University, Utrecht, the Netherlands

^2^ Department of Pharmacovigilance, Medicines Evaluation Board, Utrecht, the Netherlands

^3^ School of Pharmacy, University of Waterloo, Waterloo, Ontario, Canada

^4^ Department of Clinical Epidemiology, Leiden University Medical Centre, Leiden, the Netherlands

^5^ Department of Biomedical Data Sciences, Leiden University Medical Centre, Leiden, the Netherlands

^6^ Department of Pharmaceutical Sciences, School of Health Sciences, University of Iceland, Reykjavik, Iceland

^7^ Department of Clinical Pharmacy, University Medical Centre Utrecht, Utrecht, the Netherlands

* Both authors contributed equally.

Correspondence to: Prof. Dr. Helga Gardarsdottir, [h.gardarsdottir@uu.nl](mailto:h.gardarsdottir@uu.nl)

Journal: European Journal of Clinical Pharmacology

**Contents**

[**Supplementary tables** 3](#_Toc201917577)

[**Table S1.** List of included ATC-codes and active substances. 3](#_Toc201917578)

[**Table S2.** ATC code with DDDs of benzodiazepines/benzodiazepine-related drugs. 6](#_Toc201917579)

[**Table S3.** Most common indication categories for benzodiazepine/ benzodiazepine-related drug prescriptions in Lithuania between January 1, 2018, and December 31, 2024. 7](#_Toc201917580)

[**Table S4.** ARIMA model estimates of monthly benzodiazepine and benzodiazepine-related drug prescribing patterns stratified by sex in Lithuania from January 1, 2018, to December 31, 2024, based on electronic prescription data. 8](#_Toc201917581)

[**Table S5.** ARIMA model estimates of monthly benzodiazepine and benzodiazepine-related drug prescribing patterns stratified by age groups in Lithuania from January 1, 2018, to December 31, 2024, based on electronic prescription data. 9](#_Toc201917582)

[**Table S6.** ARIMA model estimates of monthly benzodiazepine and benzodiazepine-related drug (BZRD) prescribing patterns stratified by indication for prescription in Lithuania from January 1, 2018, to December 31, 2024, based on electronic prescription data. 11](#_Toc201917583)

[**Table S7.** ARIMA model estimates of monthly benzodiazepine and benzodiazepine-related drug prescribing patterns stratified by prescriber speciality in Lithuania from January 1, 2018, to December 31, 2024, based on electronic prescription data. 13](#_Toc201917584)

[**Table S8.** ARIMA model estimates for monthly alternative medicines prescribed for anxiety, mood disorders, and sleep disorders prescribing patterns in Lithuania from January 1, 2018, to December 31, 2024, based on electronic prescription data. 15](#_Toc201917585)

[**Supplementary figures** 17](#_Toc201917586)

[**Figure S1.** Flow charts of included benzodiazepine/benzodiazepine-related drug prescriptions and patients with a prescription for benzodiazepines and benzodiazepine-related drugs in Lithuania between January 1, 2018, and December 31, 2024. 17](#_Toc201917587)

[**Figure S2.** Schematic overview of the definitions of incidence and prevalence. 18](#_Toc201917588)

[**Figure S3.** Schematic overview of the definition of long-term use. 19](#_Toc201917589)

[**Figure S4.** Monthly trends in prevalence of benzodiazepines and benzodiazepine-related drugs stratified by sex in Lithuania from January 1, 2018, to December 31, 2024, based on electronic prescriptions. 20](#_Toc201917590)

[**Figure S5.** Monthly trends in prevalence of benzodiazepines and benzodiazepine-related drugs stratified by age groups in Lithuania from January 1, 2018, to December 31, 2024, based on electronic prescriptions. 21](#_Toc201917591)

[**Figure S6.** Monthly trends in prevalence of benzodiazepines and benzodiazepine-related drugs stratified by indication for prescription in Lithuania from January 1, 2018, to December 31, 2024, based on electronic prescriptions. 22](#_Toc201917592)

[**Figure S7.** Monthly trends in prevalence of benzodiazepines and benzodiazepine-related drugs stratified by prescriber speciality in Lithuania from January 1, 2018, to December 31, 2024, based on electronic prescriptions. 23](#_Toc201917593)

[**References** 24](#_Toc201917594)

# **Supplementary tables**

## ***Table S1.*** *List of included ATC-codes and active substances.*

| Medicine class (ATC code) | Active substance (ATC code) |
| --- | --- |
| Benzodiazepines/benzodiazepine-related drugs | |
| Anxiolytic benzodiazepines (N05BA) | Diazepam (N05BA01)  Chlordiazepoxide (N05BA02)  Medazepam (N05BA03)  Oxazepam (N05BA04)  Potassium clorazepate (N05BA05)  Lorazepam (N05BA06)  Adinazolam (N05BA07)^A^  Bromazepam (N05BA08)  Clobazam (N05BA09)  Ketazolam (N05BA10)^A^  Prazepam (N05BA11)^A^  Alprazolam (N05BA12)  Halazepam (N05BA13)^A^  Pinazepam (N05BA14)^A^  Camazepam (N05BA15)^A^  Nordazepam (N05BA16)^A^  Fludiazepam (N05BA17)^A^  Ethyl loflazepate (N05BA18)^A^  Etizolam (N05BA19)^A^  Clotiazepam (N05BA21)^A^  Cloxazolam (N05BA22)^A^  Tofisopam (N05BA023)  Bentazepam (N05BA024)^A^  Mexazolam (N05BA025)^A^  Lorazepam, combinations (N05BA026)^A^ |
| Hypnotic benzodiazepines (N05CD) | Flurazepam (N05CD01)^A^  Nitrazepam (N05CD02)  Flunitrazepam (N05CD03)^A^  Estazolam (N05CD04)  Triazolam (N05CD05)  Lormetazepam (N05CD06)^A^  Temazepam (N05CD07)^A^  Midazolam (N05CD08)  Brotizolam (N05CD09)^A^  Quazepam (N05CD010)^A^  Loprazolam (N05CD011)^A^  Doxefazepam (N05CD012)^A^  Cinolazepam (N05CD013)^A^  Remimazolam (N05CD014)  Nimetazepam (N05CD015)^A^ |
| Benzodiazepine-related drugs (N05CF) | Zopiclone (N05CF01)  Zolpidem (N05CF02)  Zaleplon (N05CF03)^A^  Eszopiclone (N05CF04) |
| Alternative medicines | |
| Gabapentinoids (N02BF)^B,C^ | Gabapentin (N02BF01)  Pregabalin (N02BF02)  Mirogabalin (N02BF03)^A^ |
| Antiepileptic benzodiazepines (N03AE) | Clonazepam (N03AE01) |
| Diazepines, oxazepines, thiazepines, and oxepines (N05AH)^B^ | Loxapine (N05AH01)  Clozapine (N05AH02)  Olanzapine (N05AH03)  Quetiapine (N05AH04)  Asenapine (N05AH05)  Clotiapine (N05AH06)^A^  Olanzapine and samidorphan (N05AH53)^A^ |
| Diphenylmethane derivatives (N05BB)^B^ | Hydroxyzine (N05BB01)^A^  Captodiame (N05BB02)^A^  Hydroxyzine, combinations (N05BB51)^A^ |
| Carbamates (N05BC)^B^ | Meprobamate (N05BC01)^A^  Emylcamate (N05BC03)^A^  Mebutamate (N05BC04)^A^  Meprobamate, combinations (N05BC51)^A^ |
| Azaspirodecanedione derivatives (N05BE)^B^ | Buspirone (N05BE01) |
| Other anxiolytics (N05BX)^B^ | Mephenoxalone (N05BX01)^A^  Gedocarnil (N05BX02)^A^  Etifoxine (N05BX03)^A^  Fabomotizole (N05BX04)^A^  Lavandulae aetheroleum (N05BX05) |
| Melatonin receptor agonists (N05CH)^B^ | Melatonin (N05CH01)  Ramelteon (N05CH02)^A^  Tasimelteon (N05CH03) |
| Non-selective monoamine reuptake inhibitors (N06AA)^B^ | Desipramine (N06AA01)^A^  Imipramine (N06AA02)^A^  Imipramine oxide (N06AA03)^A^  Clomipramine (N06AA04)  Opipramol (N06AA05)^A^  Trimipramine (N06AA06)^A^  Lofepramine (N06AA07)^A^  Dibenzepin (N06AA08)^A^  Amitriptyline (N06AA09)  Nortriptyline (N06AA10)  Protriptyline (N06AA11)^A^  Doxepin (N06AA12)^A^  Iprindole (N06AA13)^A^  Melitracen (N06AA14)^A^  Butriptyline (N06AA15)^A^  Dosulepin (N06AA16)^A^  Amoxapine (N06AA17)^A^  Dimetacrine (N06AA18)^A^  Amineptine (N06AA19)^A^  Maprotiline (N06AA21)^A^  Quinupramine (N06AA23)^A^ |
| Selective serotonin reuptake inhibitors (N06AB)^B^ | Zimeldine (N06AB02)^A^  Fluoxetine (N06AB03)  Citalopram (N06AB04)  Paroxetine (N06AB05)  Sertraline (N06AB06)  Alaproclate (N06AB07)^A^  Fluvoxamine (N06AB08)  Etoperidone (N06AB09)^A^  Escitalopram (N06AB10) |
| Monoamine oxidase inhibitors, non-selective (N06AF)^B^ | Isocarboxazid (N06AF01)^A^  Nialamide (N06AF02)^A^  Phenelzine (N06AF03)^A^  Tranylcypromine (N06AF04)^A^  Iproniazide (N06AF05)^A^  Iproclozide (N06AF06)^A^ |
| Monoamine oxidase A inhibitors (N06AG)^B^ | Moclobemide (N06AG02)^A^  Toloxatone (N06AG03)^A^ |
| Other antidepressants (N06AX)^B^ | Oxitriptan (N06AX01)  Tryptophan (N06AX02)^A^  Mianserin (N06AX03)^A^  Nomifensine (N06AX04)^A^  Trazodone (N06AX05)  Nefazodone (N06AX06)^A^  Minaprine (N06AX07)^A^  Bifemelane (N06AX08)^A^  Viloxazine (N06AX09)^A^  Oxaflozane (N06AX10)^A^  Mirtazapine (N06AX11)  Bupropion (N06AX12)  Medifoxamine (N06AX13)^A^  Tianeptine (N06AX14)  Pivagabine (N06AX15)^A^  Venlafaxine (N06AX16)  Milnacipran (N06AX17)^A^  Reboxetine (N06AX18)^A^  Gepirone (N06AX19)^A^  Duloxetine (N06AX21)  Agomelatine (N06AX22)  Desvenlafaxine (N06AX23)^A^  Vilazodone (N06AX24)^A^  Hyperici herba (N06AX25)^A^  Vortioxetine (N06AX26)  Levomilnacipran (N06AX28)^A^  Brexanolone (N06AX29)^A^  Zuranolone (N06AX31)^A^  Bupropion and Dextromethorphan (N06AX62)^A^ |

^A^Medicine is not registered in Lithuania, as of May 1, 2025 [1].

^B^In our primary analyses, we only included prescriptions of these alternative medicines prescribed for anxiety and related disorders (ICD-10 codes: F40*-F48*; F06.4); mood disorders (ICD codes: F30*-F39*; F06.3); sleep disorders (ICD codes: G47*; F51*), as these are most relevant to assess the impact of the policy measures.

^C^During our study period, gabapentinoids were moved from the N03AX group to the new N02BF group.

ATC, Anatomical Therapeutic Chemical.

## ***Table S2.*** *ATC code with DDDs of benzodiazepines/benzodiazepine-related drugs.*

| Medicine class (ATC code) | Active substance (ATC code) | 1 DDD (oral administration, mg) |
| --- | --- | --- |
| Anxiolytic benzodiazepines (N05BA) | Diazepam (N05BA01) | 10 |
|  | Chlordiazepoxide (N05BA02) | 30 |
|  | Medazepam (N05BA03) | 20 |
|  | Oxazepam (N05BA04) | 50 |
|  | Potassium clorazepate (N05BA05) | 20 |
|  | Lorazepam (N05BA06) | 2.5 |
|  | Adinazolam (N05BA07) | Not assigned |
|  | Bromazepam (N05BA08) | 10 |
|  | Clobazam (N05BA09) | 20 |
|  | Ketazolam (N05BA10) | Not assigned |
|  | Prazepam (N05BA11) | 30 |
|  | Alprazolam (N05BA12) | 1 |
|  | Halazepam (N05BA13) | 100 |
|  | Pinazepam (N05BA14) | Not assigned |
|  | Camazepam (N05BA15) | 30 |
|  | Nordazepam (N05BA16) | 15 |
|  | Fludiazepam (N05BA17) | 0.75 |
|  | Ethyl loflazepate (N05BA18) | 2 |
|  | Etizolam (N05BA19) | Not assigned |
|  | Clotiazepam (N05BA21) | Not assigned |
|  | Cloxazolam (N05BA22) | Not assigned |
|  | Tofisopam (N05BA023) | Not assigned; 50^A^ |
|  | Bentazepam (N05BA024) | 75 |
|  | Mexazolam (N05BA025) | Not assigned |
|  | Lorazepam, combinations (N05BA026) | Not assigned |
| Hypnotic benzodiazepines (N05CD) | Flurazepam (N05CD01) | 30 |
|  | Nitrazepam (N05CD02) | 5 |
|  | Flunitrazepam (N05CD03) | 1 |
|  | Estazolam (N05CD04) | 3 |
|  | Triazolam (N05CD05) | 0.25 |
|  | Lormetazepam (N05CD06) | 1 |
|  | Temazepam (N05CD07) | 20 |
|  | Midazolam (N05CD08) | 15 |
|  | Brotizolam (N05CD09) | 0.25 |
|  | Quazepam (N05CD010) | 15 |
|  | Loprazolam (N05CD11) | 1 |
|  | Doxefazepam (N05CD12) | Not assigned |
|  | Cinolazepam (N05CD13) | Not assigned |
|  | Remimazolam (N05CD14) | Not assigned |
|  | Nimetazepam (N05CD15) | Not assigned |
| Benzodiazepine-related drugs (N05CF) | Zopiclone (N05CF01) | 7.5 |
|  | Zolpidem (N05CF02) | 10 |
|  | Zaleplon (N05CF03) | 10 |
|  | Eszopiclone (N05CF04) | 2 |

^A^ Based on typical dosing regimen.

ATC, Anatomical Therapeutic Chemical; DDD, defined daily dose.

## ***Table S3.*** *Most common indication categories for benzodiazepine/ benzodiazepine-related drug prescriptions in Lithuania between January 1, 2018, and December 31, 2024.*

| Indication, n (%) | Anxiolytic benzodiazepines (N05BA),  n = 5,142,266 | Hypnotic benzodiazepines (N05CD),  n = 470,608 | Benzodiazepine-related drugs (N05CF),  n = 1,361,185 |
| --- | --- | --- | --- |
| Sleep disorders (G47*) | 656,355 (12.8) | 147,176 (31.3) | 462,959 (34.0) |
| Inorganic sleep disorders (F51*) | 345,366 (6.7) | 94,141 (20.0) | 222,458 (16.3) |
| Other anxiety disorders (F41*) | 1,119,606 (21.8) | 45,623 (9.7) | 156,634 (11.5) |
| Recurrent depressive disorder (F33*) | 611,563 (11.9) | 41,480 (8.8) | 124,739 (9.2) |
| Other mental disorders due to brain damage, dysfunction and somatic disease (F06*) | 486,170 (9.5) | 37,578 (8.0) | 93,479 (6.9) |
| Hypertensive heart disease (I11*) | 319,814 (6.2) | 17,378 (3.7) | 51,543 (3.8) |
| Depressive episode (F32*) | 209,843 (4.1) | 16,382 (3.5) | 48,918 (3.6) |
| Schizophrenia (F20*) | 182,066 (3.5) | 4,179 (0.9) | 13,733 (1.0) |
| Reactions to high stress and adaptation disorders (F43*) | 78,595 (1.5) | 4,922 (1.0) | 16,988 (1.2) |
| Vascular dementia (F01*) | 64,354 (1.3) | 4,394 (0.9) | 10,961 (0.8) |
| Other cerebrovascular diseases (I67*) | 77,752 (1.5) | 5,512 (1.2) | 10,721 (0.8) |
| Schizoaffective disorders (F25*) | 92,642 (1.8) | 2,802 (0.6) | 10,956 (0.8) |
| Symptoms and signs related to emotional state (R45*) | 71,890 (1.4) | 2,710 (0.6) | 12,974 (1.0) |
| Somatoform disorders (F45*) | 93,048 (1.8) | 4,047 (0.9) | 8,243 (0.6) |
| Epilepsy (G40*) | 64,174 (1.2) | 6,572 (1.4) | 1,180 (0.1) |
| Unspecified dementia (F03*) | 30,830 (0.6) | 1,998 (0.4) | 5,398 (0.4) |
| Other neurotic disorders (F48*) | 37,261 (0.7) | 1,495 (0.3) | 3,457 (0.3) |
| Type II diabetes (E11*) | 12,358 (0.2) | 1,369 (0.3) | 3,529 (0.3) |
| Mental and behavioural disorders due to the use of sedatives and hypnotics (F13*) | 30,444 (0.6) | 1,151 (0.2) | 3,651 (0.3) |
| Autonomic (vegetative) nervous system disorders (G90*) | 31,543 (0.6) | 916 (0.2) | 3,521 (0.3) |
| Other | 526,592 (10.2) | 28,783 (6.1) | 95,143 (7.0) |

Categories were based on first three symbols of the International Classification of Diseases (ICD-10-AM).

## ***Table S4.*** *ARIMA model estimates of monthly benzodiazepine and benzodiazepine-related drug prescribing patterns stratified by sex in Lithuania from January 1, 2018, to December 31, 2024, based on electronic prescription data.*

|  | Baseline slope [95% CI] | Step change [95% CI] | Change in slope [95% CI] |
| --- | --- | --- | --- |
| Male | | | |
| Incidence  Anxiolytic benzodiazepines (N05BA)  Hypnotic benzodiazepines (N05CD)  Benzodiazepine-related drugs (N05CF) | 0.029 [0.011; 0.047]  0.010 [0.006; 0.013]  0.017 [0.013; 0.021] | -0.062 [-0.598; 0.474]  -0.111 [-0.241; 0.020]  0.107 [-0.015; 0.229] | -0.043 [-0.068; -0.019]  -0.008 [-0.014; -0.002]  -0.022 [-0.028; -0.016] |
| Prevalence  Anxiolytic benzodiazepines (N05BA)  Hypnotic benzodiazepines (N05CD)  Benzodiazepine-related drugs (N05CF) | 0.218 [0.194; 0.241]  0.035 [0.031; 0.038]  0.075 [0.063; 0.087] | -0.521 [-1.228; 0.186]  -0.401 [-0.551; -0.251]  0.300 [-0.067; 0.666] | -0.210 [-0.242; -0.178]  -0.016 [-0.022; -0.010]  -0.056 [-0.073; -0.040] |
| Long-term use prevalence^A^  Anxiolytic benzodiazepines (N05BA)  Hypnotic benzodiazepines (N05CD)  Benzodiazepine-related drugs (N05CF) | 0.049 [0.028; 0.071]  0.007 [0.005; 0.008]  0.018 [0.011; 0.026] | 0.123 [-0.366; 0.612]  -0.048 [-0.108; 0.012]  -0.003 [-0.234; 0.228] | -0.017 [-0.044; 0.010]  0 [-0.003; 0.002]  -0.003 [-0.014; 0.008] |
| Female |  |  |  |
| Incidence  Anxiolytic benzodiazepines (N05BA)  Hypnotic benzodiazepines (N05CD)  Benzodiazepine-related drugs (N05CF) | 0.076 [0.030; 0.122]  0.014 [0.006; 0.023]  0.036 [0.027; 0.045] | -0.477 [-1.828; 0.873]  -0.214 [-0.466; 0.037]  0.083 [-0.176; 0.342] | -0.103 [-0.167; -0.039]  -0.011 [-0.023; 0.001]  -0.044 [-0.056; -0.031] |
| Prevalence  Anxiolytic benzodiazepines (N05BA)  Hypnotic benzodiazepines (N05CD)  Benzodiazepine-related drugs (N05CF) | 0.607 [0.538; 0.677]  0.078 [0.070; 0.086]  0.171 [0.143; 0.199] | -2.039 [-4.096; 0.019]  -0.895 [-1.273; -0.516]  0.657 [-0.195; 1.510] | -0.550 [-0.645; -0.455]  -0.029 [-0.044; -0.015]  -0.113 [-0.151; -0.075] |
| Long-term use prevalence^A^  Anxiolytic benzodiazepines (N05BA)  Hypnotic benzodiazepines (N05CD)  Benzodiazepine-related drugs (N05CF) | 0.162 [0.098; 0.226]  0.013 [0.008; 0.018]  0.039 [0.020; 0.057] | -0.149 [-1.436; 1.138]  -0.107 [-0.275; 0.060]  0.232 [-0.366; 0.830] | -0.069 [-0.167; 0.029]  0.005 [-0.001; 0.011]  -0.003 [-0.028; 0.022] |

The table presents 1) baseline slopes, representing monthly changes in the outcome prior to the implementation of the policy measures; 2) step changes, capturing any immediate change in the outcome at the time of implementation of the policy measures; and 3) changes in slopes, reflecting additional monthly changes in the outcome after implementation of the policy measures. Models were assessed for incidence and Prevalences across the three benzodiazepine/BZRD classes stratified by sex.

Incidence was defined as the number of patients initiating treatment with a medicine of a given class within a particular month following a minimum of 180 days without any prescription for a medicine of the same class, with the condition that patients have been enrolled in the database for at least 180 days. Prevalence referred to the total number of unique patients receiving at least one prescription for a medicine of a given class within a particular month. Long-term use was characterised as a treatment duration of at least 180 days, allowing treatment gaps of up to 30 days between prescriptions.

^A^ Long-term use was only assessed from January 1, 2019 onwards.

## ***Table S5.*** *ARIMA model estimates of monthly benzodiazepine and benzodiazepine-related drug prescribing patterns stratified by age groups in Lithuania from January 1, 2018, to December 31, 2024, based on electronic prescription data.*

|  | Baseline slope [95% CI] | Step change [95% CI] | Change in slope [95% CI] |
| --- | --- | --- | --- |
| 18-29 years |  |  |  |
| Incidence  Anxiolytic benzodiazepines (N05BA)  Hypnotic benzodiazepines (N05CD)  Benzodiazepine-related drugs (N05CF) | 0.018 [0.012; 0.023]  0.001 [0; 0.002]  0.004 [0.002; 0.006] | 0.239 [0.076; 0.402]  -0.012 [-0.038; 0.015]  0.083 [0.029; 0.136] | -0.014 [-0.021; -0.006]  -0.001 [-0.002; 0]  -0.004 [-0.007; -0.002] |
| Prevalence  Anxiolytic benzodiazepines (N05BA)  Hypnotic benzodiazepines (N05CD)  Benzodiazepine-related drugs (N05CF) | 0.061 [0.055; 0.068]  0.004 [0.002; 0.005]  0.013 [0.010; 0.017] | 0.415 [0.228; 0.603]  -0.027 [-0.077; 0.023]  0.143 [0.040; 0.246] | -0.035 [-0.045; -0.024]  -0.002 [-0.004; 0]  -0.010 [-0.014; -0.006] |
| Long-term use prevalence^A^  Anxiolytic benzodiazepines (N05BA)  Hypnotic benzodiazepines (N05CD)  Benzodiazepine-related drugs (N05CF) | 0.010 [0.006; 0.014]  0.001 [0; 0.002]  0.002 [0.001; 0.003] | 0.015 [-0.060; 0.091]  0.002 [-0.018; 0.021]  0.030 [-0.002; 0.063] | -0.001 [-0.005; 0.003]  0 [-0.001; 0]  -0.001 [-0.002; 0] |
| 30-44 years |  |  |  |
| Incidence  Anxiolytic benzodiazepines (N05BA)  Hypnotic benzodiazepines (N05CD)  Benzodiazepine-related drugs (N05CF) | 0.036 [0.024; 0.048]  0.003 [0.001; 0.005]  0.011 [0.008; 0.014] | 0.033 [-0.318; 0.384]  -0.036 [-0.088; 0.015]  0.119 [0.038; 0.199] | -0.044 [-0.060; -0.028]  -0.003 [-0.005; 0]  -0.014 [-0.018; -0.010] |
| Prevalence  Anxiolytic benzodiazepines (N05BA)  Hypnotic benzodiazepines (N05CD)  Benzodiazepine-related drugs (N05CF) | 0.155 [0.130; 0.180]  0.012 [0.010; 0.014]  0.036 [0.030; 0.042] | 0.111 [-0.631; 0.853]  -0.145 [-0.231; -0.059]  0.275 [0.091; 0.459] | -0.152 [-0.186; -0.118]  -0.007 [-0.011; -0.003]  -0.034 [-0.043; -0.026] |
| Long-term use prevalence^A^  Anxiolytic benzodiazepines (N05BA)  Hypnotic benzodiazepines (N05CD)  Benzodiazepine-related drugs (N05CF) | 0.034 [0.024; 0.044]  0.001 [0.001; 0.002]  0.008 [0.005; 0.011] | 0.352 [0.138; 0.566]  -0.013 [-0.032; 0.007]  0.033 [-0.069; 0.135] | -0.021 [-0.032; -0.011]  0 [-0.001; 0.001]  -0.003 [-0.007; 0.001] |
| 45-59 years |  |  |  |
| Incidence  Anxiolytic benzodiazepines (N05BA)  Hypnotic benzodiazepines (N05CD)  Benzodiazepine-related drugs (N05CF) | 0.076 [0.041; 0.111]  0.011 [0.005; 0.017]  0.031 [0.023; 0.039] | -0.306 [-1.344; 0.732]  -0.131 [-0.298; 0.035]  0.199 [-0.024; 0.422] | -0.104 [-0.153; -0.056]  -0.009 [-0.017; -0.001]  -0.041 [-0.052; -0.030] |
| Prevalence  Anxiolytic benzodiazepines (N05BA)  Hypnotic benzodiazepines (N05CD)  Benzodiazepine-related drugs (N05CF) | 0.403 [0.343; 0.464]  0.048 [0.042; 0.055]  0.117 [0.098; 0.136] | -1.363 [-3.155; 0.428]  -0.643 [-0.935; -0.352]  0.519 [-0.053; 1.091] | -0.399 [-0.482; -0.315]  -0.027 [-0.039; -0.015]  -0.101 [-0.127; -0.074] |
| Long-term use prevalence^A^  Anxiolytic benzodiazepines (N05BA)  Hypnotic benzodiazepines (N05CD)  Benzodiazepine-related drugs (N05CF) | 0.082 [0.056; 0.108]  0.007 [0.005; 0.010]  0.024 [0.014; 0.034] | 0.675 [0.111; 1.240]  -0.107 [-0.203; -0.011]  0.112 [-0.180; 0.404] | -0.037 [-0.065; -0.009]  0 [-0.004; 0.003]  -0.006 [-0.020; 0.009] |
| 60-74 years |  |  |  |
| Incidence  Anxiolytic benzodiazepines (N05BA)  Hypnotic benzodiazepines (N05CD)  Benzodiazepine-related drugs (N05CF) | 0.109 [0.039; 0.179]  0.024 [0.010; 0.037]  0.056 [0.041; 0.071] | -1.002 [-3.049; 1.044]  -0.383 [-0.765; -0.002]  0.038 [-0.385; 0.461] | -0.157 [-0.254; -0.060]  -0.018 [-0.036; -0.001]  -0.069 [-0.090; -0.049] |
| Prevalence  Anxiolytic benzodiazepines (N05BA)  Hypnotic benzodiazepines (N05CD)  Benzodiazepine-related drugs (N05CF) | 0.807 [0.681; 0.932]  0.121 [0.109; 0.133]  0.275 [0.232; 0.319] | -4.035 [-7.726; -0.344]  -1.485 [-2.034; -0.936]  0.448 [-0.884; 1.780] | -0.785 [-0.959; -0.612]  -0.049 [-0.070; -0.027]  -0.202 [-0.261; -0.143] |
| Long-term use prevalence^A^  Anxiolytic benzodiazepines (N05BA)  Hypnotic benzodiazepines (N05CD)  Benzodiazepine-related drugs (N05CF) | 0.230 [0.144; 0.316]  0.019 [0.013; 0.026]  0.069 [0.037; 0.100] | -0.355 [-2.261; 1.551]  -0.154 [-0.387; 0.079]  -0.182 [-1.134; 0.769] | -0.130 [-0.261; 0.002]  0.006 [-0.002; 0.015]  -0.014 [-0.059; 0.031] |
| ≥75 years |  |  |  |
| Incidence  Anxiolytic benzodiazepines (N05BA)  Hypnotic benzodiazepines (N05CD)  Benzodiazepine-related drugs (N05CF) | 0.059 [-0.044; 0.163]  0.054 [0.032; 0.077]  0.074 [0.054; 0.093] | -0.718 [-3.794; 2.358]  -0.704 [-1.597; 0.190]  0.098 [-0.474; 0.670] | -0.131 [-0.273; 0.012]  -0.047 [-0.085; -0.008]  -0.092 [-0.119; -0.064] |
| Prevalence  Anxiolytic benzodiazepines (N05BA)  Hypnotic benzodiazepines (N05CD)  Benzodiazepine-related drugs (N05CF) | 1.530 [1.353; 1.707]  0.228 [0.205; 0.252]  0.487 [0.378; 0.595] | -4.858 [-10.116; 0.399]  -2.264 [-3.328; -1.200]  0.213 [-3.264; 3.69] | -1.373 [-1.614; -1.131]  -0.079 [-0.121; -0.037]  -0.271 [-0.470; -0.072] |
| Long-term use prevalence^A^  Anxiolytic benzodiazepines (N05BA)  Hypnotic benzodiazepines (N05CD)  Benzodiazepine-related drugs (N05CF) | 0.421 [0.220; 0.622]  0.043 [0.029; 0.058]  0.111 [0.047; 0.176] | -0.225 [-4.073; 3.624]  -0.206 [-0.722; 0.311]  0.318 [-1.385; 2.022] | -0.134 [-0.436; 0.168]  0.017 [-0.001; 0.036]  0.007 [-0.090; 0.103] |

The table presents 1) baseline slopes, representing monthly changes in the outcome prior to the implementation of the policy measures; 2) step changes, capturing any immediate change in the outcome at the time of implementation of the policy measures; and 3) changes in slopes, reflecting additional monthly changes in the outcome after implementation of the policy measures. Models were assessed for incidence and Prevalences across the three benzodiazepine/BZRD classes stratified by age groups. Note that stratified analyses for the age groups <18 years were not feasible due to limited numbers in specific subgroups.

Incidence was defined as the number of patients initiating treatment with a medicine of a given class within a particular month following a minimum of 180 days without any prescription for a medicine of the same class, with the condition that patients have been enrolled in the database for at least 180 days. Prevalence referred to the total number of unique patients receiving at least one prescription for a medicine of a given class within a particular month. Long-term use was characterised as a treatment duration of at least 180 days, allowing treatment gaps of up to 30 days between prescriptions.

^A^ Long-term use was only assessed from January 1, 2019 onwards.

## ***Table S6.*** *ARIMA model estimates of monthly benzodiazepine and benzodiazepine-related drug (BZRD) prescribing patterns stratified by indication for prescription in Lithuania from January 1, 2018, to December 31, 2024, based on electronic prescription data.*

|  | Baseline slope [95% CI] | Step change [95% CI] | Change in slope [95% CI] |
| --- | --- | --- | --- |
| Sleep disorders |  |  |  |
| Incidence  Anxiolytic benzodiazepines (N05BA)  Hypnotic benzodiazepines (N05CD)  Benzodiazepine-related drugs (N05CF) | 0.012 [0.006; 0.018]  0.009 [0.006; 0.012]  0.017 [0.013; 0.022] | -0.096 [-0.271; 0.080]  -0.127 [-0.259; 0.005]  0.080 [-0.055; 0.216] | -0.012 [-0.020; -0.004]  -0.007 [-0.012; -0.002]  -0.016 [-0.023; -0.010] |
| Prevalence  Anxiolytic benzodiazepines (N05BA)  Hypnotic benzodiazepines (N05CD)  Benzodiazepine-related drugs (N05CF) | 0.077 [0.061; 0.092]  0.028 [0.023; 0.033]  0.064 [0.053; 0.075] | -0.271 [-0.735; 0.194]  -0.336 [-0.577; -0.094]  0.339 [-0.081; 0.759] | -0.053 [-0.074; -0.032]  -0.005 [-0.015; 0.004]  -0.023 [-0.041; -0.006] |
| Long-term use prevalence^A^  Anxiolytic benzodiazepines (N05BA)  Hypnotic benzodiazepines (N05CD)  Benzodiazepine-related drugs (N05CF) | 0.013 [0.006; 0.020]  0.004 [0.002; 0.006]  0.010 [0.006; 0.015] | 0.135 [-0.027; 0.297]  -0.056 [-0.128; 0.015]  0.097 [-0.060; 0.254] | 0.002 [-0.006; 0.009]  0.004 [0.001; 0.007]  0.007 [0.001; 0.012] |
| Anxiety and related disorders |  |  |  |
| Incidence  Anxiolytic benzodiazepines (N05BA)  Hypnotic benzodiazepines (N05CD)  Benzodiazepine-related drugs (N05CF) | 0.021 [0.010; 0.033]  0.001 [0; 0.003]  0.003 [0.002; 0.003] | -0.104 [-0.447; 0.239]  -0.021 [-0.058; 0.016]  -0.011 [-0.037; 0.015] | -0.033 [-0.048; -0.018]  -0.001 [-0.003; 0]  -0.004 [-0.006; -0.003] |
| Prevalence  Anxiolytic benzodiazepines (N05BA)  Hypnotic benzodiazepines (N05CD)  Benzodiazepine-related drugs (N05CF) | 0.122 [0.095; 0.149]  0.008 [0.007; 0.010]  0.017 [0.013; 0.021] | -0.157 [-0.975; 0.662]  -0.086 [-0.157; -0.016]  0.035 [-0.088; 0.158] | -0.113 [-0.150; -0.076]  -0.004 [-0.007; -0.001]  -0.015 [-0.021; -0.009] |
| Long-term use prevalence^A^  Anxiolytic benzodiazepines (N05BA)  Hypnotic benzodiazepines (N05CD)  Benzodiazepine-related drugs (N05CF) | 0.019 [0.008; 0.030]  0.002 [0.001; 0.002]  0.005 [0.003; 0.007] | 0.182 [-0.075; 0.438]  -0.014 [-0.031; 0.003]  0.030 [-0.037; 0.097] | 0.001 [-0.011; 0.013]  0.001 [0; 0.001]  -0.001 [-0.003; 0.002] |
| Mood disorders |  |  |  |
| Incidence  Anxiolytic benzodiazepines (N05BA)  Hypnotic benzodiazepines (N05CD)  Benzodiazepine-related drugs (N05CF) | -0.002 [-0.005; 0.001]  0 [-0.001; 0.002]  0.001 [0; 0.002] | -0.004 [-0.084; 0.077]  -0.015 [-0.053; 0.023]  -0.004 [-0.032; 0.024] | 0.001 [-0.003; 0.005]  0 [-0.001; 0.002]  -0.001 [-0.003; 0] |
| Prevalence  Anxiolytic benzodiazepines (N05BA)  Hypnotic benzodiazepines (N05CD)  Benzodiazepine-related drugs (N05CF) | 0.078 [0.069; 0.086]  0.009 [0.007; 0.011]  0.019 [0.016; 0.022] | -0.067 [-0.315; 0.181]  -0.092 [-0.178; -0.007]  0.053 [-0.029; 0.135] | -0.069 [-0.081; -0.057]  -0.004 [-0.007; 0]  -0.015 [-0.019; -0.011] |
| Long-term use prevalence^A^  Anxiolytic benzodiazepines (N05BA)  Hypnotic benzodiazepines (N05CD)  Benzodiazepine-related drugs (N05CF) | 0.023 [0.015; 0.031]  0.002 [0.001; 0.002]  0.006 [0.004; 0.008] | 0.219 [0.033; 0.405]  -0.007 [-0.027; 0.013]  0.050 [-0.026; 0.126] | -0.010 [-0.018; -0.001]  0.001 [0; 0.001]  -0.001 [-0.004; 0.001] |
| Other mental disorders |  |  |  |
| Incidence  Anxiolytic benzodiazepines (N05BA)  Hypnotic benzodiazepines (N05CD)  Benzodiazepine-related drugs (N05CF) | 0.002 [-0.001; 0.005]  0 [0; 0.001]  0.001 [0.001; 0.002] | 0.046 [-0.039; 0.131]  -0.007 [-0.023; 0.009]  0.010 [0.003; 0.016] | -0.002 [-0.005; 0.002]  0 [-0.001; 0]  -0.002 [-0.002; -0.001] |
| Prevalence  Anxiolytic benzodiazepines (N05BA)  Hypnotic benzodiazepines (N05CD)  Benzodiazepine-related drugs (N05CF) | 0.053 [0.046; 0.060]  0.004 [0.003; 0.005]  0.009 [0.008; 0.010] | 0.057 [-0.140; 0.254]  -0.039 [-0.076; -0.001]  0.069 [0.025; 0.113] | -0.042 [-0.051; -0.033]  -0.001 [-0.003; 0]  -0.006 [-0.007; -0.004] |
| Long-term use prevalence^A^  Anxiolytic benzodiazepines (N05BA)  Hypnotic benzodiazepines (N05CD)  Benzodiazepine-related drugs (N05CF) | 0.017 [0.011; 0.022]  0.001 [0; 0.002]  0.002 [0.002; 0.003] | 0.171 [0.053; 0.288]  -0.007 [-0.026; 0.013]  0.030 [-0.003; 0.063] | -0.007 [-0.013; -0.002]  0 [0; 0.001]  0 [-0.001; 0.002] |
| Other disorders |  |  |  |
| Incidence  Anxiolytic benzodiazepines (N05BA)  Hypnotic benzodiazepines (N05CD)  Benzodiazepine-related drugs (N05CF) | 0.023 [0.015; 0.030]  0.002 [0.001; 0.003]  0.005 [0.004; 0.006] | -0.366 [-0.585; -0.146]  -0.036 [-0.062; -0.009]  -0.041 [-0.079; -0.003] | -0.024 [-0.034; -0.014]  -0.001 [-0.003; 0]  -0.006 [-0.008; -0.004] |
| Prevalence  Anxiolytic benzodiazepines (N05BA)  Hypnotic benzodiazepines (N05CD)  Benzodiazepine-related drugs (N05CF) | 0.095 [0.084; 0.107]  0.008 [0.007; 0.009]  0.017 [0.015; 0.020] | -1.260 [-1.577; -0.942]  -0.141 [-0.194; -0.088]  -0.130 [-0.203; -0.058] | -0.097 [-0.113; -0.082]  -0.005 [-0.007; -0.003]  -0.017 [-0.021; -0.013] |
| Long-term use prevalence^A^  Anxiolytic benzodiazepines (N05BA)  Hypnotic benzodiazepines (N05CD)  Benzodiazepine-related drugs (N05CF) | 0.009 [0.006; 0.012]  0.001 [0.001; 0.002]  0.002 [0.001; 0.003] | -0.013 [-0.082; 0.057]  -0.017 [-0.031; -0.002]  -0.006 [-0.033; 0.020] | -0.005 [-0.008; -0.001]  0 [-0.001; 0.001]  -0.001 [-0.002; 0] |

The table presents 1) baseline slopes, representing monthly changes in the outcome prior to the implementation of the policy measures; 2) step changes, capturing any immediate change in the outcome at the time of implementation of the policy measures; and 3) changes in slopes, reflecting additional monthly changes in the outcome after implementation of the policy measures. Models were assessed for incidence and Prevalences across the three benzodiazepine/BZRD classes stratified by indication for prescription.

Incidence was defined as the number of patients initiating treatment with a medicine of a given class within a particular month following a minimum of 180 days without any prescription for a medicine of the same class, with the condition that patients have been enrolled in the database for at least 180 days. Prevalence referred to the total number of unique patients receiving at least one prescription for a medicine of a given class within a particular month. Long-term use was characterised as a treatment duration of at least 180 days, allowing treatment gaps of up to 30 days between prescriptions.

^A^ Long-term use was only assessed from January 1, 2019 onwards.

## ***Table S7.*** *ARIMA model estimates of monthly benzodiazepine and benzodiazepine-related drug prescribing patterns stratified by prescriber speciality in Lithuania from January 1, 2018, to December 31, 2024, based on electronic prescription data.*

|  | Baseline slope [95% CI] | Step change [95% CI] | Change in slope [95% CI] |
| --- | --- | --- | --- |
| General practitioner |  |  |  |
| Incidence  Anxiolytic benzodiazepines (N05BA)  Hypnotic benzodiazepines (N05CD)  Benzodiazepine-related drugs (N05CF) | 0.049 [0.026; 0.073]  0.012 [0.009; 0.016]  0.022 [0.016; 0.027] | -0.704 [-1.416; 0.007]  -0.205 [-0.359; -0.051]  -0.102 [-0.253; 0.050] | -0.061 [-0.092; -0.029]  -0.010 [-0.016; -0.004]  -0.022 [-0.029; -0.015] |
| Prevalence  Anxiolytic benzodiazepines (N05BA)  Hypnotic benzodiazepines (N05CD)  Benzodiazepine-related drugs (N05CF) | 0.263 [0.217; 0.309]  0.039 [0.037; 0.041]  0.094 [0.080; 0.108] | -2.544 [-3.919; -1.169]  -0.649 [-0.763; -0.536]  -0.373 [-0.960; 0.214] | -0.227 [-0.29; -0.165]  -0.013 [-0.018; -0.009]  -0.054 [-0.079; -0.029] |
| Long-term use prevalence^A^  Anxiolytic benzodiazepines (N05BA)  Hypnotic benzodiazepines (N05CD)  Benzodiazepine-related drugs (N05CF) | 0.036 [0.020; 0.051]  0.006 [0.004; 0.009]  0.015 [0.010; 0.020] | 0.124 [-0.225; 0.474]  -0.097 [-0.175; -0.019]  0.041 [-0.137; 0.219] | -0.001 [-0.018; 0.016]  0.003 [0; 0.006]  0.004 [-0.003; 0.010] |
| Psychiatrist |  |  |  |
| Incidence  Anxiolytic benzodiazepines (N05BA)  Hypnotic benzodiazepines (N05CD)  Benzodiazepine-related drugs (N05CF) | 0 [-0.004; 0.005]  0.001 [0; 0.003]  0.003 [0.002; 0.005] | 0.153 [0.021; 0.284]  -0.016 [-0.07; 0.037]  0.064 [0.022; 0.107] | -0.003 [-0.009; 0.003]  -0.001 [-0.003; 0.001]  -0.005 [-0.007; -0.003] |
| Prevalence  Anxiolytic benzodiazepines (N05BA)  Hypnotic benzodiazepines (N05CD)  Benzodiazepine-related drugs (N05CF) | 0.134 [0.117; 0.151]  0.015 [0.011; 0.019]  0.033 [0.026; 0.039] | 0.855 [0.368; 1.342]  -0.054 [-0.215; 0.108]  0.439 [0.246; 0.632] | -0.120 [-0.144; -0.096]  -0.005 [-0.012; 0.001]  -0.025 [-0.034; -0.015] |
| Long-term use prevalence^A^  Anxiolytic benzodiazepines (N05BA)  Hypnotic benzodiazepines (N05CD)  Benzodiazepine-related drugs (N05CF) | 0.058 [0.047; 0.069]  0.003 [0.001; 0.004]  0.010 [0.005; 0.014] | 0.295 [-0.114; 0.704]  0.008 [-0.038; 0.053]  0.141 [-0.029; 0.311] | -0.034 [-0.048; -0.019]  0.002 [0; 0.003]  0.001 [-0.005; 0.007] |
| Internal medicine physician |  |  |  |
| Incidence  Anxiolytic benzodiazepines (N05BA)  Hypnotic benzodiazepines (N05CD)  Benzodiazepine-related drugs (N05CF) | 0.006 [0.001; 0.011]  0.001 [0; 0.002]  0.003 [0.002; 0.004] | -0.173 [-0.335; -0.012]  -0.037 [-0.068; -0.006]  -0.026 [-0.055; 0.004] | -0.009 [-0.015; -0.002]  -0.001 [-0.002; 0]  -0.004 [-0.005; -0.003] |
| Prevalence  Anxiolytic benzodiazepines (N05BA)  Hypnotic benzodiazepines (N05CD)  Benzodiazepine-related drugs (N05CF) | 0.041 [0.031; 0.05]  0.007 [0.006; 0.008]  0.013 [0.01; 0.016] | -0.586 [-0.872; -0.300]  -0.134 [-0.185; -0.082]  -0.07 [-0.172; 0.032] | -0.043 [-0.056; -0.03]  -0.004 [-0.005; -0.002]  -0.01 [-0.015; -0.006] |
| Long-term use prevalence^A^  Anxiolytic benzodiazepines (N05BA)  Hypnotic benzodiazepines (N05CD)  Benzodiazepine-related drugs (N05CF) | 0.006 [0.003; 0.009]  0.001 [0; 0.002]  0.003 [0.002; 0.003] | -0.01 [-0.085; 0.065]  -0.016 [-0.029; -0.004]  0.005 [-0.016; 0.026] | -0.002 [-0.005; 0.002]  0 [0; 0.001]  -0.001 [-0.002; 0] |
| Neurologist |  |  |  |
| Incidence  Anxiolytic benzodiazepines (N05BA)  Hypnotic benzodiazepines (N05CD)  Benzodiazepine-related drugs (N05CF) | 0.001 [0; 0.002]  0 [0; 0.001]  0.001 [0; 0.002] | 0.099 [0.078; 0.121]  0.007 [-0.002; 0.017]  0.040 [0.018; 0.062] | -0.001 [-0.002; 0]  0 [-0.001; 0]  -0.001 [-0.002; 0] |
| Prevalence  Anxiolytic benzodiazepines (N05BA)  Hypnotic benzodiazepines (N05CD)  Benzodiazepine-related drugs (N05CF) | 0.004 [0.002; 0.005]  0 [0; 0.001]  0.002 [0.001; 0.003] | 0.179 [0.128; 0.231]  0.012 [0; 0.024]  0.056 [0.018; 0.093] | -0.004 [-0.006; -0.002]  0 [-0.001; 0]  -0.002 [-0.003; 0] |
| Long-term use prevalence^A^  Anxiolytic benzodiazepines (N05BA)  Hypnotic benzodiazepines (N05CD)  Benzodiazepine-related drugs (N05CF) | 0.001 [0.001; 0.002]  0 [0; 0]  0 [0; 0] | 0.003 [-0.006; 0.013]  0.001 [0.001; 0.001]  0.002 [-0.002; 0.006] | -0.001 [-0.001; -0.001]  0 [-0.001; 0.001]  0 [0; 0] |
| Other speciality |  |  |  |
| Incidence  Anxiolytic benzodiazepines (N05BA)  Hypnotic benzodiazepines (N05CD)  Benzodiazepine-related drugs (N05CF) | 0.003 [0.002; 0.005]  0 [0; 0.001]  0.001 [0.001; 0.002] | -0.008 [-0.059; 0.043]  -0.009 [-0.02; 0.003]  0.007 [-0.008; 0.022] | -0.004 [-0.006; -0.002]  0 [0; 0]  -0.001 [-0.002; 0] |
| Prevalence  Anxiolytic benzodiazepines (N05BA)  Hypnotic benzodiazepines (N05CD)  Benzodiazepine-related drugs (N05CF) | 0.015 [0.011; 0.019]  0.002 [0.002; 0.003]  0.005 [0.004; 0.006] | -0.106 [-0.217; 0.005]  -0.031 [-0.051; -0.011]  -0.013 [-0.057; 0.03] | -0.011 [-0.016; -0.006]  0 [-0.001; 0]  -0.002 [-0.004; 0] |
| Long-term use prevalence^A^  Anxiolytic benzodiazepines (N05BA)  Hypnotic benzodiazepines (N05CD)  Benzodiazepine-related drugs (N05CF) | 0.002 [0.001; 0.003]  0 [NaN; NaN]  0.001 [0; 0.001] | 0.001 [-0.026; 0.028]  -0.007 [NaN; NaN]  0.001 [-0.011; 0.012] | 0.001 [-0.001; 0.002]  0 [0; 0]  0.001 [0; 0.001] |

The table presents 1) baseline slopes, representing monthly changes in the outcome prior to the implementation of the policy measures; 2) step changes, capturing any immediate change in the outcome at the time of implementation of the policy measures; and 3) changes in slopes, reflecting additional monthly changes in the outcome after implementation of the policy measures. Models were assessed for incidence and Prevalences across the three benzodiazepine/BZRD classes stratified by prescriber speciality.

Incidence was defined as the number of patients initiating treatment with a medicine of a given class within a particular month following a minimum of 180 days without any prescription for a medicine of the same class, with the condition that patients have been enrolled in the database for at least 180 days. Prevalence referred to the total number of unique patients receiving at least one prescription for a medicine of a given class within a particular month. Long-term use was characterised as a treatment duration of at least 180 days, allowing treatment gaps of up to 30 days between prescriptions.

^A^ Long-term use was only assessed from January 1, 2019 onwards.

## ***Table S8.*** *ARIMA model estimates for monthly* *alternative medicines prescribed for anxiety, mood disorders, and sleep disorders prescribing patterns in Lithuania from January 1, 2018, to December 31, 2024, based on electronic prescription data.*

|  | Baseline slope [95% CI] | Step change [95% CI] | Change in slope [95% CI] |
| --- | --- | --- | --- |
| Sleep disorders, anxiety and related disorders, and mood disorders | | | |
| Incidence  Gabapentinoids (N02BF)  Antiepileptic benzodiazepines (N03AE)  Diazepines, oxazepanes, thiazepines,  and oxepines (N05AH)  Azaspirodecanedione derivatives  (N05BE)  Non-selective monoamine reuptake  inhibitors (N06AA)  SSRIs (N06AB)  Other antidepressants (N06AX) | 0 [0; 0.001]  -0.001 [-0.003; 0]  0.006 [0.002; 0.009]  0 [0; 0.001]  0 [-0.001; 0]  -0.006 [-0.009; -0.002]  -0.004 [-0.006; -0.001] | 0.008 [0.002; 0.015]  -0.019 [-0.073; 0.036]  0.087 [-0.009; 0.183]  -0.005 [-0.018; 0.008]  0.008 [-0.007; 0.022]  0.175 [0.082; 0.268]  0.137 [0.069; 0.205] | 0 [-0.001; 0]  0 [-0.003; 0.002]  -0.004 [-0.009; 0.001]  0 [-0.001; 0]  0 [-0.001; 0]  0.005 [0; 0.010]  0.002 [-0.001; 0.006] |
| Prevalence  Gabapentinoids (N02BF)  Antiepileptic benzodiazepines (N03AE)  Diazepines, oxazepanes, thiazepines,  and oxepines (N05AH)  Azaspirodecanedione derivatives  (N05BE)  Non-selective monoamine reuptake  inhibitors (N06AA)  SSRIs (N06AB)  Other antidepressants (N06AX) | 0.001 [0.001; 0.002]  0.017 [0.012; 0.022] 0.070 [0.062; 0.077]  0.001 [0.001; 0.002]  0.008 [0.007; 0.010]  0.102 [0.092; 0.111]  0.076 [0.062; 0.090] | 0.02 [0.001; 0.038]  -0.112 [-0.273; 0.048]  0.094 [-0.110; 0.298]  -0.007 [-0.014; 0.001]  -0.048 [-0.089; -0.007]  -0.158 [-0.420; 0.104]  -0.117 [-0.508; 0.274] | 0.002 [0.001; 0.003]  -0.019 [-0.027; -0.011]  -0.031 [-0.041; -0.020]    -0.001 [-0.001; -0.001]  -0.009 [-0.011; -0.007]  -0.070 [-0.084; -0.057]  -0.053 [-0.073; -0.034] |
| Sleep disorders | | | |
| Incidence  Gabapentinoids (N02BF)  Antiepileptic benzodiazepines (N03AE)  Diazepines, oxazepanes, thiazepines,  and oxepines (N05AH)  Non-selective monoamine reuptake  inhibitors (N06AA)  SSRIs (N06AB)  Other antidepressants (N06AX) | NA  0.001 [0; 0.001]  0.004 [0.003; 0.006]  0 [0; 0.001]  0 [0; 0.001]  0.001 [0; 0.001] | NA  -0.026 [-0.048; -0.003]  0.054 [0.001; 0.106]  0.003 [-0.001; 0.007]  -0.001 [-0.004; 0.003]  0.016 [0.004; 0.028] | NA  -0.001 [-0.002; 0]  -0.002 [-0.005; 0.001]  0 [0; 0]  0 [0; 0]  -0.001 [-0.001; 0] |
| Prevalence  Gabapentinoids (N02BF)  Antiepileptic benzodiazepines (N03AE)  Diazepines, oxazepanes, thiazepines,  and oxepines (N05AH)  Non-selective monoamine reuptake  inhibitors (N06AA)  SSRIs (N06AB)  Other antidepressants (N06AX) | NA  0.009 [0.007; 0.011]  0.018 [0.015; 0.021]  0.001 [0; 0.001]  0.001 [0; 0.001]  0.003 [0.002; 0.004] | NA  -0.078 [-0.139; -0.016]  0.078 [-0.026; 0.181]  0.001 [-0.004; 0.006]  0.001 [-0.003; 0.006]  0.039 [0.017; 0.062] | NA  -0.009 [-0.012; -0.007]  0.001 [-0.004; 0.006]  -0.001 [-0.001; 0]  0 [0; 0]  0 [-0.001; 0.001] |
| Anxiety and related disorders | | | |
| Incidence  Gabapentinoids (N02BF)  Antiepileptic benzodiazepines (N03AE)  Diazepines, oxazepanes, thiazepines,  and oxepines (N05AH)  Azaspirodecanedione derivatives  (N05BE)  Non-selective monoamine reuptake  inhibitors (N06AA)  SSRIs (N06AB)  Other antidepressants (N06AX) | NA  0 [-0.001; 0]  0.003 [0.001; 0.004]  0 [-0.001; 0.002]  0 [-0.001; 0.001]  0.005 [0.003; 0.006]  0.002 [0.002; 0.003] | NA  -0.008 [-0.026; 0.010]  0.022 [-0.014; 0.057]  -0.001 [-0.015; 0.013]  0.005 [0.002; 0.009]  0.039 [0.002; 0.076]  0.028 [0.004; 0.051] | NA  0 [-0.001; 0]  -0.003 [-0.005; -0.001]  0 [-0.001; 0]  0 [-0.001; 0]  -0.003 [-0.005; -0.001]  -0.002 [-0.004; -0.001] |
| Prevalence  Gabapentinoids (N02BF)  Antiepileptic benzodiazepines (N03AE)  Diazepines, oxazepanes, thiazepines,  and oxepines (N05AH)  Azaspirodecanedione derivatives  (N05BE)  Non-selective monoamine reuptake  inhibitors (N06AA)  SSRIs (N06AB)  Other antidepressants (N06AX) | 0 [0; 0.001]  0.005 [0.003; 0.007]  0.016 [0.014; 0.018]  0 [0; 0.001]  0.002 [0.002; 0.002]  0.029 [0.025; 0.032]  0.016 [0.014; 0.019] | 0.008 [-0.001; 0.016]  -0.021 [-0.075; 0.034]  0.079 [0.018; 0.139]  0.001 [-0.004; 0.007]  0.001 [-0.009; 0.010]  0.193 [0.095; 0.291]  0.120 [0.041; 0.200] | 0.001 [0.001; 0.001]  -0.006 [-0.008; -0.004]  -0.007 [-0.010; -0.004]  0 [-0.001; 0]  -0.002 [-0.002; -0.001]  -0.005 [-0.009; 0]  -0.004 [-0.007; 0] |
| Mood disorders | | | |
| Incidence  Gabapentinoids (N02BF)  Antiepileptic benzodiazepines (N03AE)  Diazepines, oxazepanes, thiazepines,  and oxepines (N05AH)  Azaspirodecanedione derivatives  (N05BE)  Non-selective monoamine reuptake  inhibitors (N06AA)  SSRIs (N06AB)  Other antidepressants (N06AX) | 0 [0; 0.001]  -0.002 [-0.003; -0.001]  0 [-0.001; 0.001]  0 [0; 0.001]  0 [-0.001; 0]  -0.011 [-0.014; -0.008]  -0.007 [-0.009; -0.005] | 0.004 [0.001; 0.008]  0.017 [-0.004; 0.037]  -0.004 [-0.032; 0.023]  -0.008 [-0.015; -0.002]  -0.001 [-0.013; 0.011]  0.133 [0.058; 0.209]  0.091 [0.039; 0.143] | 0 [-0.001; 0]  0.001 [0.001; 0.002]  0 [-0.001; 0.001]  0 [-0.001; 0]  0 [0; 0.001]  0.009 [0.005; 0.013]  0.005 [0.003; 0.008] |
| Prevalence  Gabapentinoids (N02BF)  Antiepileptic benzodiazepines (N03AE)  Diazepines, oxazepanes, thiazepines,  and oxepines (N05AH)  Azaspirodecanedione derivatives  (N05BE)  Non-selective monoamine reuptake  inhibitors (N06AA)  SSRIs (N06AB)  Other antidepressants (N06AX) | 0.001 [0; 0.001]  0.003 [0.001; 0.004]  0.036 [0.034; 0.039]  0.001 [0; 0.002]  0.006 [0.005; 0.007]  0.073 [0.056; 0.090]  0.059 [0.041; 0.076] | 0.014 [0.005; 0.023]  0.006 [-0.034; 0.046]  -0.058 [-0.130; 0.015]  -0.009 [-0.026; 0.008]  -0.05 [-0.083; -0.017]  -0.292 [-0.758; 0.175]  -0.208 [-0.669; 0.252] | 0.001 [0; 0.001]  -0.004 [-0.005; -0.002]  -0.025 [-0.029; -0.021]  -0.001 [-0.001; 0]  -0.007 [-0.008; -0.005]  -0.065 [-0.090; -0.041]  -0.051 [-0.076; -0.025] |

The table presents 1) baseline slopes, representing monthly changes in the outcome prior to the implementation of the policy measures; 2) step changes, capturing any immediate change in the outcome at the time of implementation of the policy measures; and 3) changes in slopes, reflecting additional monthly changes in the outcome after implementation of the policy measures. Models were assessed for incidences and prevalences.

Incidence was defined as the number of patients initiating treatment with a medicine of a given class within a particular month following a minimum of 180 days without any prescription for a medicine of the same class, with the condition that patients have been enrolled in the database for at least 180 days. Prevalence referred to the total number of unique patients receiving at least one prescription for a medicine of a given class within a particular month.

There were no electronic prescriptions issued for melatonin receptor agonists (N05CH) and other anxiolytics (N05BX) prior to the introduction of the policy measures, and thus, the impact of the policy measures on incidence and prevalence could not be evaluated. Additionally, diphenylmethane derivatives (N05BB), carbamates (N05BC), monoamine oxidase inhibitors, non-selective (N06AF), and monoamine oxidase A inhibitors (N06AG) were neither prescribed electronically nor distributed to community pharmacies during the study period as they were not registered in Lithuania.

#

# **Supplementary figures**

## ***Figure S1.*** *Flow charts of included benzodiazepine/benzodiazepine-related drug prescriptions and patients with a prescription for benzodiazepines and benzodiazepine-related drugs in Lithuania between January 1, 2018, and December 31, 2024.*


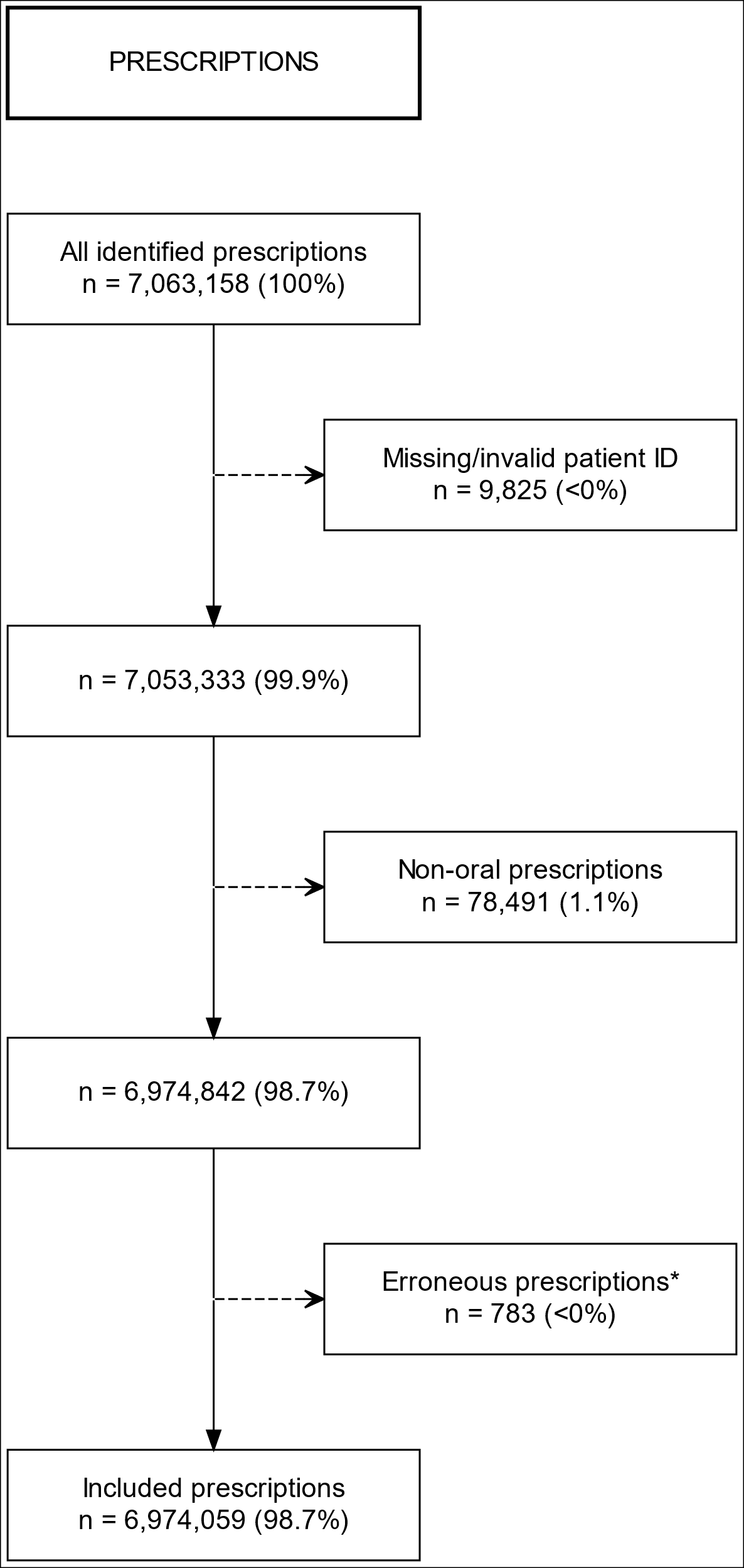

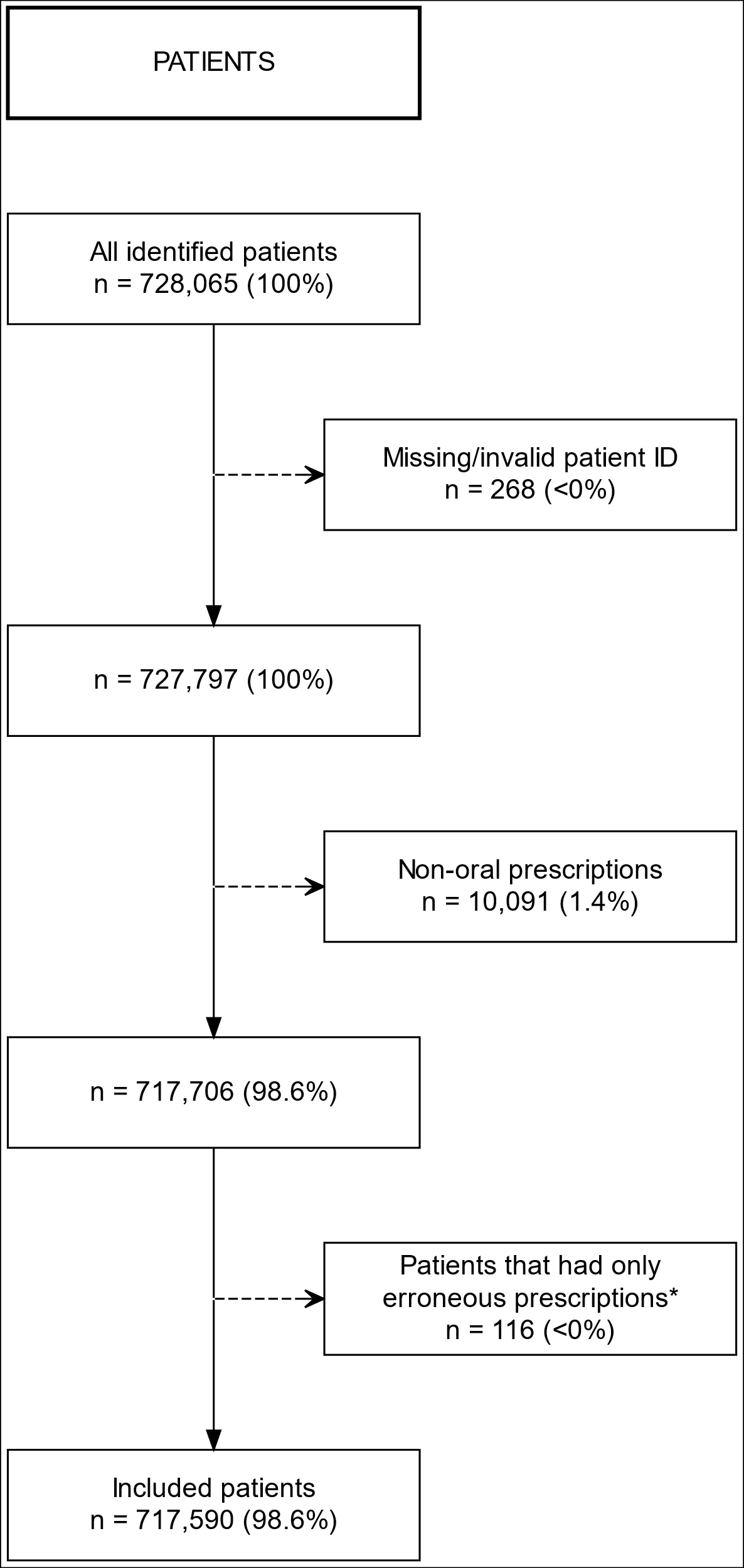


*Erroneous prescriptions were treatment duration (treatment duration of 0 or >360 days) and quantity values (missing strength or > 4 DDDs per day). Erroneous prescriptions were excluded only if these could not be corrected using prior prescription data or assumption of 1 DDD per day. Patients were excluded if they had only erroneous prescriptions. The prescription values were adjusted based on previous prescription data or 1 DDD per day for 16,585 prescriptions (no adjustments were made for the remaining 6,957,474 prescriptions).

## ***Figure S2.*** *Schematic overview of the definitions of incidence and prevalence.*


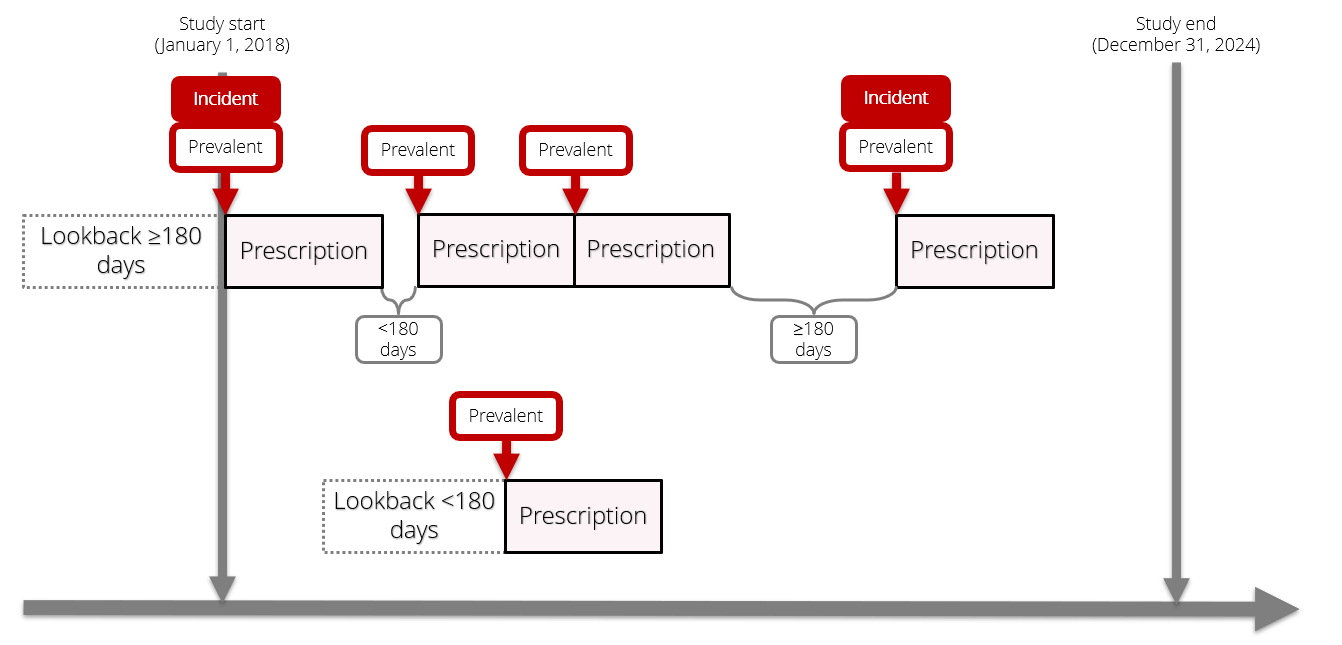


Incidence was defined as the number of patients initiating treatment with a medicine of a given class within a particular month following a minimum of 180 days without use of the medicine of the same class, with the condition that patients have been enrolled in the database for at least 180 days. Prevalence referred to the total number of unique patients receiving at least one prescription for a medicine of a given class within a particular month.

## ***Figure S3.*** *Schematic overview of the definition of long-term use.*


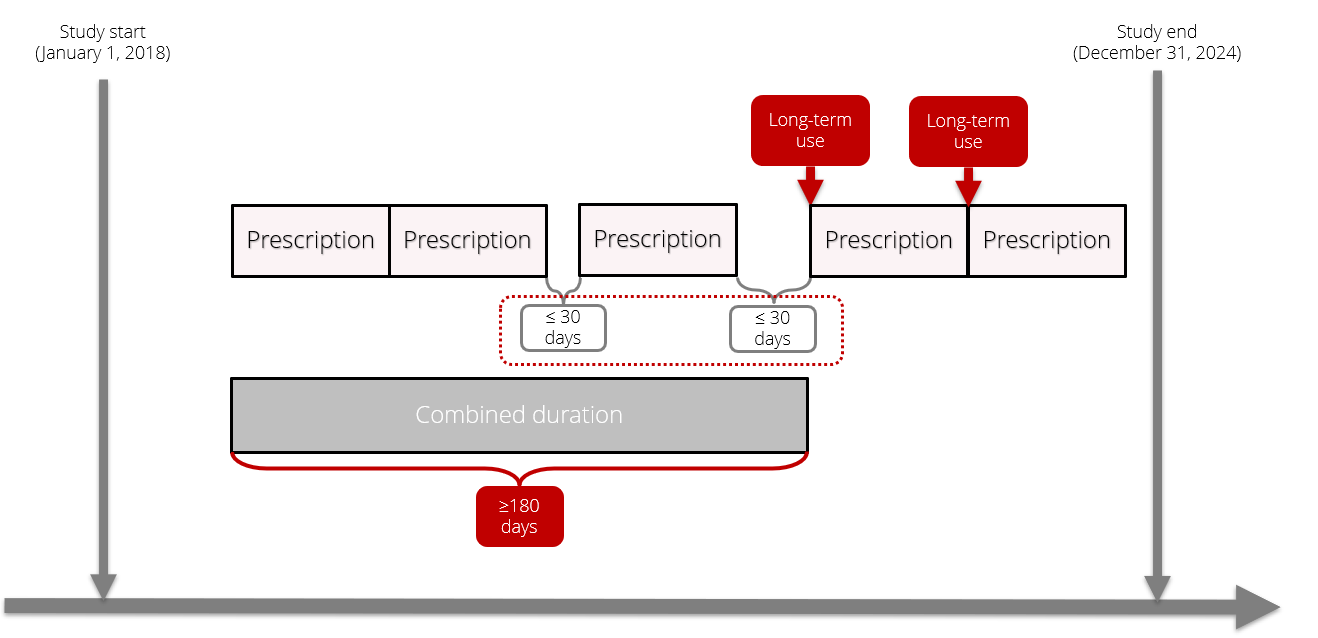


Long-term use was characterised as a treatment duration of at least 180 days, allowing treatment gaps of up to 30 days between prescriptions.

## ***Figure S4.*** *Monthly trends in prevalence of benzodiazepines and benzodiazepine-related drugs stratified by sex in Lithuania from January 1, 2018, to December 31, 2024, based on electronic prescriptions.*


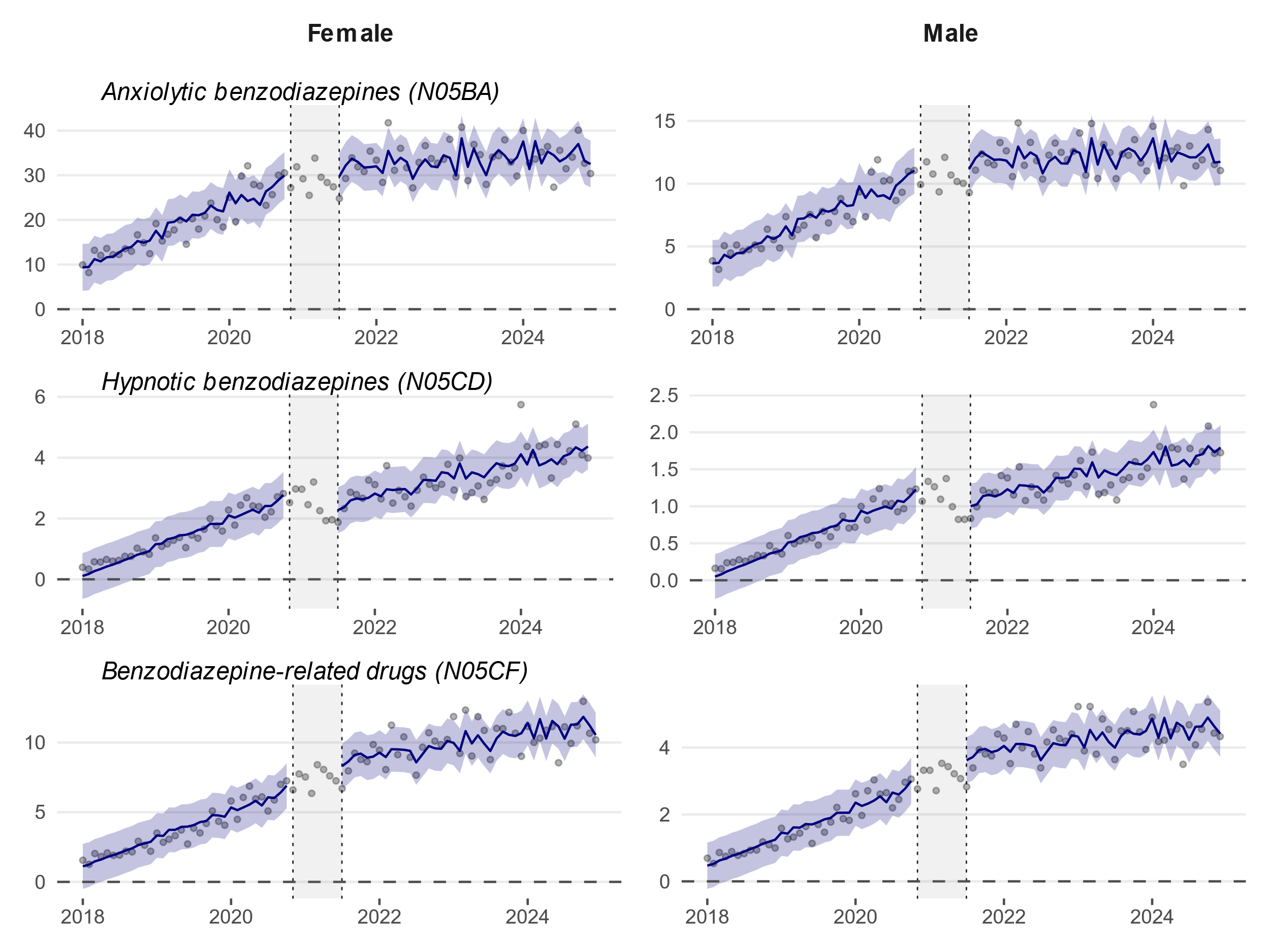


Lines represent modelled estimates with their 95% confidence intervals derived from ARIMA models, whilst points indicate monthly rates based on observed data in electronic prescription records.

Prevalence referred to the number of unique patients receiving at least one prescription for a medicine of a given class within a particular month.

## ***Figure S5.*** *Monthly trends in prevalence of benzodiazepines and benzodiazepine-related drugs stratified by age groups in Lithuania from January 1, 2018, to December 31, 2024, based on electronic prescriptions.*


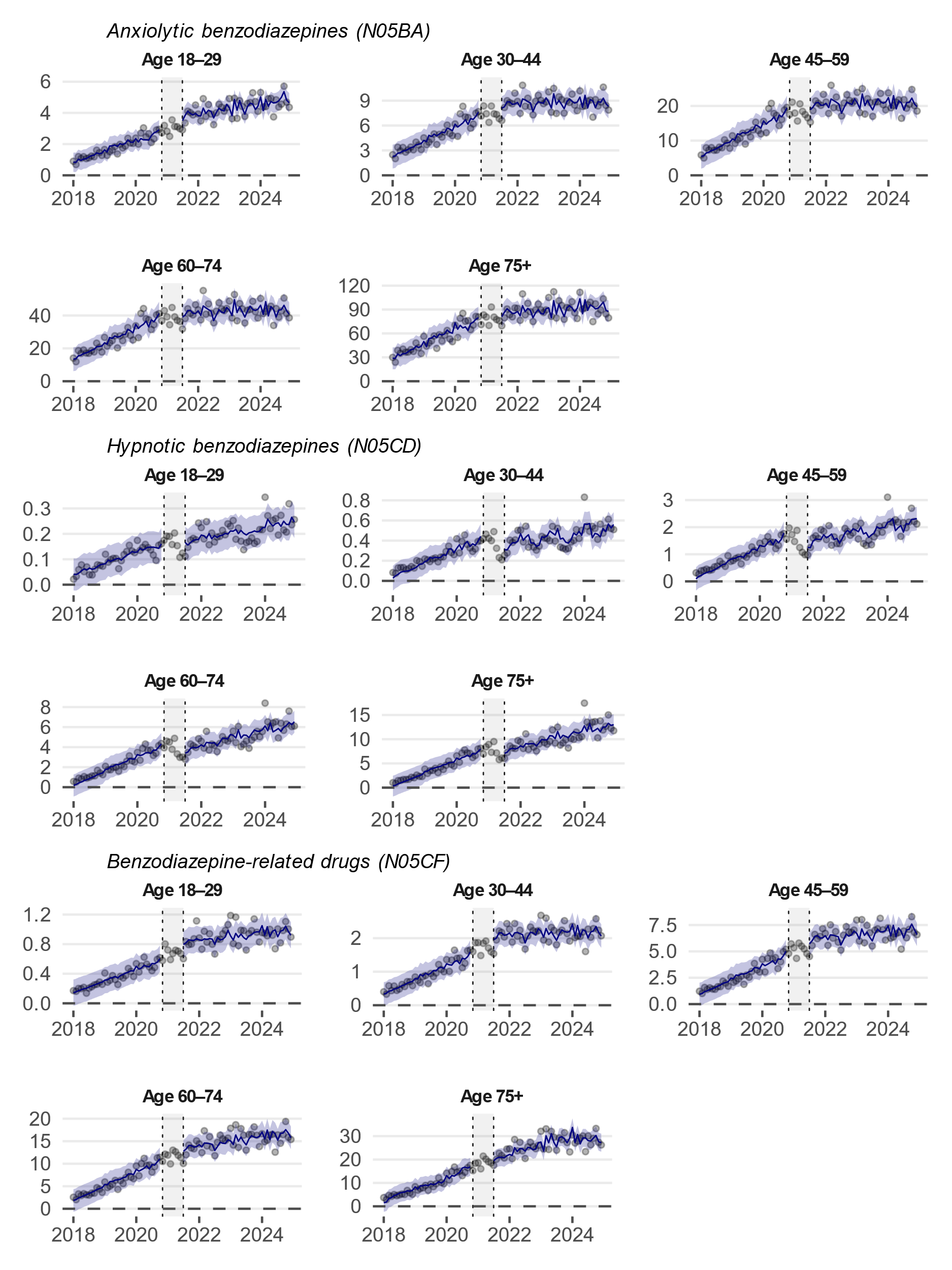


Lines represent modelled estimates with their 95% confidence intervals derived from ARIMA models, whilst points indicate monthly rates based on observed data in electronic prescription records. Note that stratified analyses for the age groups <18 years were not feasible due to limited numbers in specific subgroups.

Prevalence referred to the number of unique patients receiving at least one prescription for a medicine of a given class within a particular month.

## ***Figure S6.*** *Monthly trends in prevalence of benzodiazepines and benzodiazepine-related drugs stratified by indication for prescription in Lithuania from January 1, 2018, to December 31, 2024, based on electronic prescriptions.*


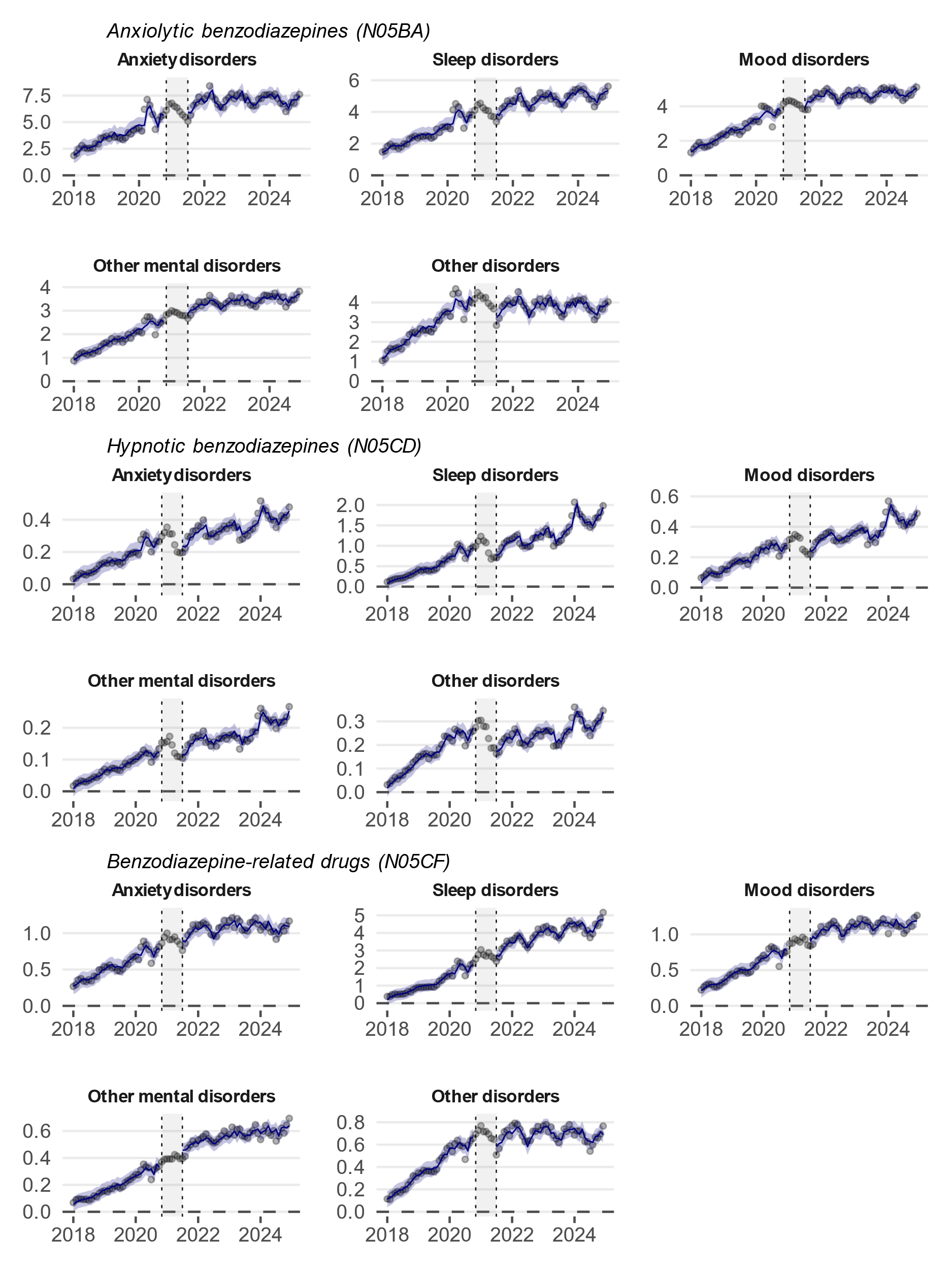


Lines represent modelled estimates with their 95% confidence intervals derived from ARIMA models, whilst points indicate monthly rates based on observed data in electronic prescription records.

Prevalence referred to the number of unique patients receiving at least one prescription for a medicine of a given class within a particular month.

## ***Figure S7.*** *Monthly trends in prevalence of benzodiazepines and benzodiazepine-related drugs stratified by prescriber speciality in Lithuania from January 1, 2018, to December 31, 2024, based on electronic prescriptions.*


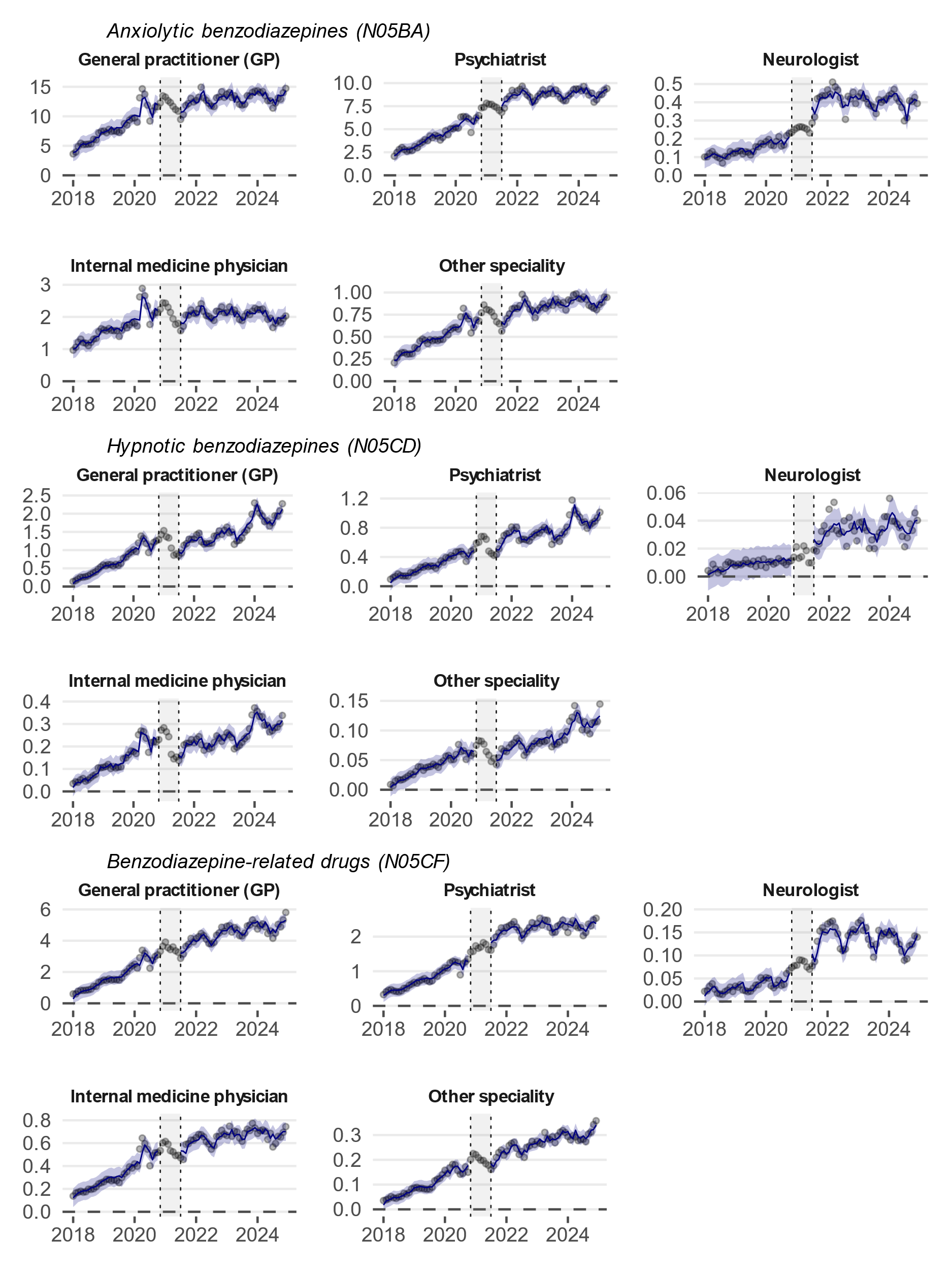


Lines represent modelled estimates with their 95% confidence intervals derived from ARIMA models, whilst points indicate monthly rates based on observed data in electronic prescription records.

Prevalence referred to the number of unique patients receiving at least one prescription for a medicine of a given class within a particular month.

# **References**

1. Lithuanian State Medicines Control Agency. VVKT - Vaistai. <https://vapris.vvkt.lt/vvkt-web/public/medications> Accessed 21 May 2025.
